# Deciphering the Ubiquitin-Mediated Pathway in Apicomplexan Parasites: A Potential Strategy to Interfere with Parasite Virulence

**DOI:** 10.1371/journal.pone.0002386

**Published:** 2008-06-11

**Authors:** Nadia Ponts, Jianfeng Yang, Duk-Won Doug Chung, Jacques Prudhomme, Thomas Girke, Paul Horrocks, Karine G. Le Roch

**Affiliations:** 1 Department of Cell Biology and Neurosciences, University of California at Riverside, Riverside, California, United States of America; 2 Center for Plant Cell Biology (CEPCEB), University of California at Riverside, Riverside, California, United States of America; 3 Department of Medicine, Institute for Science and Technology in Medicine, Keele University, Keele, United Kingdom; 4 Department of Life Sciences, Institute for Science and Technology in Medicine, Keele University, Keele, United Kingdom; Pasteur Institute, France

## Abstract

**Background:**

Reversible modification of proteins through the attachment of ubiquitin or ubiquitin-like modifiers is an essential post-translational regulatory mechanism in eukaryotes. The conjugation of ubiquitin or ubiquitin-like proteins has been demonstrated to play roles in growth, adaptation and homeostasis in all eukaryotes, with perturbation of ubiquitin-mediated systems associated with the pathogenesis of many human diseases, including cancer and neurodegenerative disorders.

**Methodology/Principal Findings:**

Here we describe the use of an HMM search of functional Pfam domains found in the key components of the ubiquitin-mediated pathway necessary to activate and reversibly modify target proteins in eight apicomplexan parasitic protozoa for which complete or late-stage genome projects exist. In parallel, the same search was conducted on five model organisms, single-celled and metazoans, to generate data to validate both the search parameters employed and aid paralog classification in *Apicomplexa*. For each of the 13 species investigated, a set of proteins predicted to be involved in the ubiquitylation pathway has been identified and demonstrates increasing component members of the ubiquitylation pathway correlating with organism and genome complexity. Sequence homology and domain architecture analyses facilitated prediction of apicomplexan-specific protein function, particularly those involved in regulating cell division during these parasite's complex life cycles.

**Conclusions/Significance:**

This study provides a comprehensive analysis of proteins predicted to be involved in the apicomplexan ubiquitin-mediated pathway. Given the importance of such pathway in a wide variety of cellular processes, our data is a key step in elucidating the biological networks that, in part, direct the pathogenicity of these parasites resulting in a massive impact on global health. Moreover, apicomplexan-specific adaptations of the ubiquitylation pathway may represent new therapeutic targets for much needed drugs against apicomplexan parasites.

## Introduction

Apicomplexans are obligate protozoa intracellular parasites responsible for several major human diseases prevalent in the developing world. These include organisms belonging to the genera *Plasmodium*, *Toxoplasma* and *Cryptosporium*. *Toxoplasma gondii* and *Cryptosporium parvum* are the etiological agents of toxoplasmosis and cryptosporidiosis, respectively, which are predominantly opportunistic infectious agents responsible for severe mortality amongst immuno-suppressed patients such as those infected with HIV. The human malarial parasite *Plasmodium falciparum*, which is responsible for over a million deaths annually [1], is perhaps the most significant apicomplexan parasitic organism. The global impact, both in terms of mortality and morbidity, of apicomplexan parasites is currently on the rise, principally due to the increase of drug resistant strains. For example, *P. falciparum* has evolved resistance to many front-line antimalarial drugs [2] and with apparently limited prospects in the delivery of new safe, effective and cheap antimalarial drugs, little immediate respite is likely. There is clearly an urgent need to characterize and validate new drug targets, effective not only against *P. falciparum* but other apicomplexan parasites as well.

Genome sequencing projects are available for several apicomplexan parasites, with many of them completed. The full genome sequence of the human malarial parasite *P. falciparum* and the rodent malaria parasites *P. yoelii*, *P. berghei* and *P. chabaudi* have been published [3], [4], with that of the human malarial parasite *P. vivax* well underway. In addition, the complete annotated genomes of *T. gondii*, *C. parvum* and *Cryptosporium hominis* have been recently released [5], [6]. Post genomic technologies, such as comparative bioinformatic approaches, global microarray and proteome analyses have created a vast amount of information pertaining to gene and protein sequence/structure prediction, interspecies identification of ortholog or paralog genes as well as temporal and developmentally associated patterns of mRNA and protein accumulation [7]–[13]. Together these studies have greatly advanced our understanding of gene expression throughout these parasites' complex life cycles in various host cells and insect vectors. Moreover, comparative analyses may provide key data regarding protein networks, and their potential as novel drug targets. For example, apicomplexan parasites, unlike higher eukaryotes, utilize the non-mevalonate pathway to synthesise isoprenoids [14]. Inhibitors of one of the key initial enzymes in this pathway, 1-deoxy-D-xylulose 5-phosphate, such as the herbicide fosmidomycin in combination with clindamycin, are currently being evaluated for treatment of uncomplicated *P. falciparum* malaria [15]. These data indicate the importance of comparative genomics in evaluating the potential for novel drug targets in apicomplexan parasites.

Here we describe a comparative analysis of one of the essential post-translational regulatory networks commonly found in eukaryotic cells–the ubiquitin/proteasome system (UPS). Modification of proteins *via* covalent conjugation to ubiquitin (or more often polyubiquitin chains) is a well-established signal for proteosomal destruction [16]. In the early 1980s, the key role of ubiquitin in the selective pathway for degradation of proteins was demonstrated, which was followed over the next two decades by additional roles in a wide range of cellular processes. In addition to ubiquitin, ubiquitin-like proteins (UBLps) have also been identified as modifiers of cellular processes [17], [18]. Together, ubiquitin and UBLps provide a reversible modification that regulates a wide range of cellular activities including DNA repair, transcription, cellular division, endocytosis, intracellular trafficking and the immune response. Importantly, defects in this pathway are associated with human diseases, including cancer and neurodegenerative disorders such as Parkinson's disease. By targeting disease-specific components of the UPS, several potential new drugs for cancer and neurodegenerative are currently under development. The potential to chemically target the UPS in the treatment of *P. falciparum* has been established [19]–[21]. However, this work focuses on the inhibition of the proteasome and the therapeutic window between the apicomplexa and the host proteasomes may be limited. Inhibition of apicomplexan-specific components of the enzymatic cascade that process, activate and transfer ubiquitin and UBLps to their various protein targets may offer attractive alternative targets.

**Figure 1:**
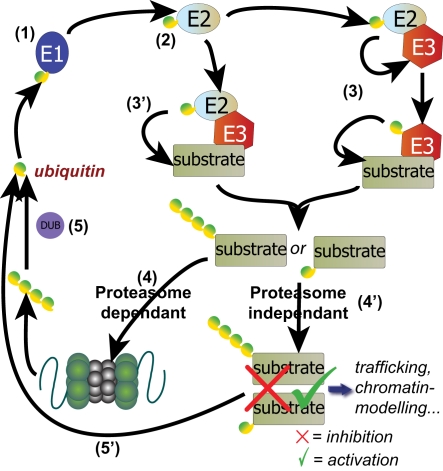
Representation of the ubiquitin-mediated pathways. (1) Ubiquitin is activated by E1 ubiquitin-activating enzyme, and (2) transferred to E2 ubiquitin-conjugating enzyme. Then, ubiquitin is either transferred to a monomeric E3 ubiquitin ligase that catalyzes ubiquitylation of the target substrate (3), or ubiquitinated E2 forms associates with the E3 to catalyze ubiquitylation of the substrate (3′). Polyubiquitinated substrate can be targeted to the proteasome and destroyed (4). Poly or monoubiquitylation can also be an activation/repression signal (4′) that modulates the substrate activity in several cellular processes such as trafficking or chromatin modeling. Finally, deubiquitinating enzymes (DUB) finally recycle ubiquitin proteins (5 and 5′).

Specificity in the conjugation of ubiquitin and ubls to their final target is elegantly achieved *via* an activation and transfer cascade [22] (figure 1). Ubiquitin-activating enzymes (termed E1) exist for ubiquitin and each UBLp. These typically adenylate the terminal glycine residue of ubiquitin/UBLp and transfers it to an internal cysteine residue with the formation of a thioester bond. The activated ubiquitin/UBLp is trans-esterified to an ubiquitin conjugating protein (termed E2). Whereas several E2 proteins are capable of accepting an activated ubiquitin molecule, typically only one E2 exists for each of the UBLps characterized thus far. Finally, ubiquitin ligases (termed E3) catalyze the transfer of ubiquitin/ubl from E2 to a lysine side chain on a specific target protein (this may occur directly or indirectly *via* conjugation to the E3) to form an isopeptide bond. Since ubiquitin contains several lysine residues, it can itself be ubiquitinylated, leading to the formation of polyubiquitin chains. Differences in affinity for ubiquitin/UBLp by the component parts of the cascade, as well as a hierarchical increase in the numbers of these proteins (*e.g.* there is one E1 for ubiquitin, several E2s and an increasing number of characterized E3s), drive the transfer of ubiquitin/UBLp through the cascade with the final target specificity mediated through the E3 complex.

Ubiquitin, a highly conserved 76 amino acid peptide, was first described in 1974 [16]. From the late 1970's onwards, a number of UBLps have also been described. These proteins do not share extensive primary sequence homology with ubiquitin, but rather share a common tertiary structure (the ubiquitin fold) and activation/conjugation mechanism through variant E1, E2 and E3 proteins. To date, within mammalian systems, over 10 UBLps have been described, including interferon-stimulated gene 15 (ISG-15), neuronal precursor cell expressed developmentally down regulated 8 (NEDD8), and small ubiquitin-related modifier (SUMO) [23].

Analysis of the E1-activating enzymes indicates that they share sequence homology to MoeB/ThiF domains of prokaryotic biosynthetic proteins involved in sulphur donor systems [24]. These proteins similarly rely on an initial adenylation of a peptide with a C-teminal diglycine motif. E1 proteins either have two MoeB/ThiF domains necessary for the adenylation and subsequent internal transfer to form a thiolester bond or are a complex of two heterodimers that each contains one MoeB/ThiF domain. Selection of an E2 for transfer the UBL modifier *via* a transesterification reaction relies on additional motifs present in E1. The E2 protein contains a single motif that mediates interaction with both E1 and E3, signifying the “shuttle” status of E2 in the transfer of ubiquitin/UBLps between activation and subsequent ligation to their final target. E2s are present as multiple isoforms, each with distinct roles. E2s exist for each UBL modifier, with multiple E2s capable of accepting ubiquitin. However, even within the ubiquitin E2 isoforms, there is functional divergence in the specific E3s they interact with, and thus the cellular processes they are involved in. For example, Rad6p and Cdc34p E2 isoforms deliver ubiquitin to E3s that ultimately target proteins involved in the regulation of DNA repair and cell cycle progression, respectively [25].

E3 ubiquitin ligases are very diverse. They have been classified into three main classes according to the presence of specific domain motifs: Homologous to E6-associated protein C-terminus (HECT), Really Interesting New Proteins (RING, *e.g.* MDM2 known to target p53) and U-box. Two sub-classes of RINGs have further been defined: RING in between RING-RING (RIR) and Cullin-RING ligases (CRL), which are multi-protein complex E3s (see [26] for a review). These CRLs are associated with proteins carrying F-box domains, which are involved in substrate recognition (see [27] for a review). The CRL anaphase-promoting complex, involved in cell cycle progression, is a typical example of an SCF-type ligase (Skp1-Cullin-Fbox). Except for the HECT family that has a direct role in catalyzing ubiquitylation, E3s are adaptors molecules that bring the E2 enzyme and the target substrate into close proximity to promote ubiquitylation. The RING finger family represents the largest group of E3s and is characterized by a cysteine/histidine-rich/zinc chelating domain that specifically promotes protein-protein interaction, as well as protein-DNA binding. In eukaryotes, RING fingers have been shown to be the key regulator of polyubiquitylation and protein degradation. However, they have also been shown to play a pivotal role in monoubiquitylation of substrates, independent of degradation. Monoubiquitylation has been shown to regulate events such as the endocytosis of cell receptors (*e.g.* the ring finger c-Cbl is required for the endocytosis of the Epidermal Growth factor Receptor, EGFR), DNA-repair (ubiquitylation of p53 by the RING finger MDM2) and transcriptional regulation (activation of NF-kB by the RING finger TRAF6).

Conversely, de-ubiquitinylation enzymes (deubiquitinases or DUBs) specifically remove ubiquitin/UBLps. DUBs are a large group of cysteine proteases or zinc-dependent metalloproteases that specifically cleave after the terminal carbonyl of the last residue of ubiquitin adducts. Compared to the proteins involved in the activation, conjugation and ligation of ubiquitin/UBLps relatively little is known about the functional role of DUBs. However, evidence suggests that DUBs are key regulators of the ubiquitin system; DUBs are functionally similar to protein phosphatases in the phosphorylation system. Based on their sequences similarities, structural studies and potential mechanism of action, DUBs fall into at least six distinct subfamilies: the ubiquitin C-terminal hydrolases (UCH-Peptidase_C12), the ubiquitin specific proteases (USP-UCH), otubains (OTU), the ataxin-3/Josephin ubiquitin protease (MJD), the JAMM isopeptidase (Mov34) and the recent *in silico* prediction of the permuted papain fold peptidase (PPPDE) [28], [29]. In addition to these DUBs subfamilies, three distinct families of deubiquitinating-like enzymes (DUBLs) are detected in eukaryotes: the SUMO-specific proteases (SENPs-Peptidase_C48), the autophagins (Peptidase_C54), and the newly predicted WLM family of zinc-dependant peptidases (WLM) mostly found in plants and fungi but apparently absent in animals [29].

The UPS is known to play important roles in modulation of immune and inflammatory responses. Deregulation of the UPS can lead to the development of inflammatory and autoimmune diseases, such as inflammatory arthritis, psoriasis, allergy and asthma (see [30] for review). Proteasome inhibitors have been developed as therapeutic molecules, principally as anticancer drugs [31]–[34]. In the context of host-pathogen interactions, both bacteria and viruses were shown to use components of their UPS as virulence factors. The E3 ubiquitin ligase from *Pseudomonas syringae* has been shown to induce sensitivity in tomato plants by targeting a host kinase, Fen, to the proteasome, which leads to the inhibition of the Fen-activated immunity-associated programmed cell death [35]. The DUB SseL (from *Salmonella enterica*), which causes gastroenteritis in humans, has similarly been implicated in its virulence [36]. Components of the UPS have been shown to be involved in many aspects of viral pathogenesis (see [37] for a review). Two RING-finger E3 ubiquitin ligases, K3 and K5, from herpes virus promote immune evasion by targeting MHC class 1 to ubiquitylation and endolysosomal degradation. The human papillomavirus E6 protein interacts with the cellular E3 ubiquitin ligase E6-associated protein. This complex mediates the proteasome-dependant degradation of the key tumor suppressor protein p53. DUBs have also been shown to be involved in viral pathogenesis. In Epstein-Barr virus infection of B cells, a group of cellular DUBs are activated, which include UCH-L1 and UCH-L5. In adenovirus infection, the viral proteinase L3 23K is responsible for the cleavage of viral precursor polyproteins, and may function as a DUB [38].

These data implicate the UPS in roles from colonization, infection, immune evasion and virulence for a range of pathogens. To date, potential roles for the UPS in mediating similar roles for apicomplexan parasites have yet to be explored. Here we describe an *in silico* proteomic analysis of UPS from eight apicomplexan parasites: *P. falciparum*, *P. vivax*, *P. yoelii*, *P. berghei*, *P. chabaudi*, *C. parvum*, *C. hominis*, and *T. gondii*. Five other eukaryotic model organisms, including *Homo sapiens*, *Saccharomyces cerevisiae*, *Caenorhabditis elegans*, *Drosophila melanogaster* and *Arabidopsis thaliana*, were analyzed in parallel for comparative purposes. We aim here to identify and describe the most complete ubiquitylation pathway in apicomplexan parasites, with a particular focus on *Plasmodium falciparum*, highlighting those components that are specific to apicomplexan parasites. Our results open new research perspectives and are expected to pilot the development of new strategies in the battle against these devastating apicomplexan diseases.

## Results and Discussion

### *In silico* prediction of ubiquitylation pathway components in apicomplexan genomes

We selected 24 Pfam domains that are known to be related to the UPS (see materials and methods section). These 24 Pfam domains are commonly found in ubiquitin and UBLps, E1 and E1-like enzymes, E2 enzymes, E3 enzymes and DUBs. Each Pfam domain family was used in an hmmsearch application of the translated genomes of *Plasmodium spp. falciparum*, *vivax*, *yoelii*, *berghei* and *chabaudi*, *T. gondii*, *Cryptosporidium spp. parvum* and *hominis*, *S. cerevisiae*, *C. elegans*, *D. melanogaster*, *H. sapiens* and *A. thaliana*. HMM searches were run using a series of increasingly stringent threshold E-values, from E-value ≤1 to E-value ≤0.1 (data not shown). With regards to the five eukaryotic model organisms that were used, the threshold E-value ≤0.5 gave the most consistent results when compared to previously published results. The number of UPS-related proteins in *A. thaliana* and the other model organisms has previously been analyzed, particularly the number of E2 and E3 enzymes that are found in *A. thaliana*, *H. sapiens*, *C. elegans* and *S. cerevisiae* (see [39]–[42] for reviews). The observation that our results (table 1) are consistent with these existing data sets would appear to validate both the HMM search strategy with a threshold E-value set at 0.5, as well as providing standard datasets in these model organisms for subsequent comparative analysis of the *Apicomplexa* data sets.

**Table 1:** Predicted number of UPS components in the 13 analyzed genomes.

Domains/Genomes	*P. falciparum*	*P. vivax*	*P. yoelii*	*P. chabaudi*	*P. berghei*	*T. gondii*	*C. parvum*	*C. hominis*	*S. cerevisiae*	*C. elegans*	*D. melanogaster*	*H. sapiens*	*A. thaliana*
**Ubiquitin and Ubiquitin like**													
**Ubiquitin**	6	6	3	2	3	6	7	5	7	8	10	25	31
**APG12**	1	0	0	0	0	0	0	0	1	1	1	1	2
**MAP1_LC3**	1	1	1	1	1	1	1	1	1	2	2	11	9
**UPF0185**	0	0	0	0	0	1	1	1	0	1	0	1	1
**Urm1**	1	1	1	1	1	1	1	1	1	0	1	2	1
**Ubiquitin activating enzymes**													
**ThiF**	8	8	8	9	9	11	8	6	8	8	10	16	14
**UBACT**													
**Ubiquitin conjugating enzymes**													
**UQ_con**	14	13	11	13	15	13	11	8	14	23	47	57	43
**Ubiquitin ligases**													
**RING finger & RING-like**	42	40	34	36	34	55	50	46	43	162	221	451	490
**HECT**	4	4	4	5	4	8	4	3	5	9	20	38	7
**cullin**	2	2	2	1	2	3	3	3	4	6	11	10	6
**U-box**	3	3	3	3	3	2	2	2	2	5	9	15	63
**F-box**	3	2	0	0	0	4	3	0	12	400	44	94	620
**De-ubiquitinases**													
**OTU**	3	3	1	0	1	10	2	1	2	5	6	15	12
**Josephin**	2	2	1	1	2	1	1	1	0	2	1	6	2
**Mov34**	6	6	6	4	5	7	4	3	4	8	11	19	15
**DUF862**	3	3	3	3	3	3	2	2	0	1	2	3	10
**WLM**	1	1	2	1	1	1	0	0	1	0	0	0	2
**UCH**	9	9	9	8	7	12	9	8	18	26	42	93	46
**Peptidase_C12**	2	2	2	2	2	2	2	2	1	4	5	4	17
**Peptidase_C48**	2	2	2	2	2	3	2	2	2	5	9	7	59
**Peptidase_C54**	1	1	1	0	0	1	1	1	1	2	2	15	2
**Total**	**114**	**109**	**94**	**92**	**95**	**145**	**114**	**96**	**127**	**678**	**454**	**883**	**1452**

Amongst the 13 proteomes investigated in this study, a total of 4453 proteins were identified as carrying one or more of the 24 selected Pfam domains (table 1 and supplemental Table S1). For example, 114 proteins were found in *P. falciparum*, 145 in *T. gondii,* 114 in *C. parvum* and 127 in *S. cerevisiae*. In each case, these numbers of UPS component proteins represent approximately 2.5% of their respective proteomes. Given the good correlation between numbers of proteins identified in each apicomplexan with that of the single celled model eukaryote *S. cerevisiae*, these data would appear to suggest that the apicomplexan datasets are relatively complete. For those *Plasmodium* species such as *P. chabaudi* or *P. berghei*, the relative under-representation of identified proteins would more likely reflect the completeness of the respective genome project rather than an absolute reduction in UPS components. The number of UPS components increases considerably in multi-cellular organisms with increased genome complexity (*e.g.* 678 proteins were identified in *C. elegans* and 883 in *H. sapiens* while some 1452 proteins were identified in *A. thaliana*). In *H. sapiens*, 162 DUBs/DUBLs were found although a previous publication only identified 95 putative DUBs/DUBLs from which 79 exhibited conserved catalytic residues [28]. Such a difference can be explained by the fact that proteomes extracted from *H. sapiens* and *D. melanogaster* genomes contain multiple isoforms for some families of DUBs and DUBLs. Furthermore, the Hidden Markov Model that we used to search for UPS components compiled more complete datasets than many other search approaches would do. For example, while our HMM search identified domain OTU-carrying proteins in apicomplexan parasites (OTU is a major sub-class of DUB) none was reported in a recent publication on parasitic protozoa deconjugating enzymes where the authors used a more selective BLASTP homology search [43]. This observation further highlights the exhaustiveness of the HMM search.

With regards to the relative abundance of each domain family, a striking observation is that a high proportion of F-box-carrying proteins are present in multi-cellular organisms (*e.g.* 43% in *A. thaliana*) while only few of them were identified in apicomplexan organisms. F-box-containing proteins are adaptor proteins in Cullin-RING-Ligase complexes (CRLs), and are involved in direct and specific substrate recognition. Previous authors have hypothesized that the very high number of F-box proteins in *A. thaliana* suggests that plants can assemble numerous CRLs, which could control a wide array of substrates [27]. The low number of F-box proteins detected in apicomplexan parasites could indicate that there is no need for these specific adaptors, or that their amino acid sequences are highly divergent from other eukaryotic cells and could not be detected using our standard HMM search. An alternate hypothesis is that a different family of proteins in apicomplexa could carry out the role of adaptor.

**Figure 2:**
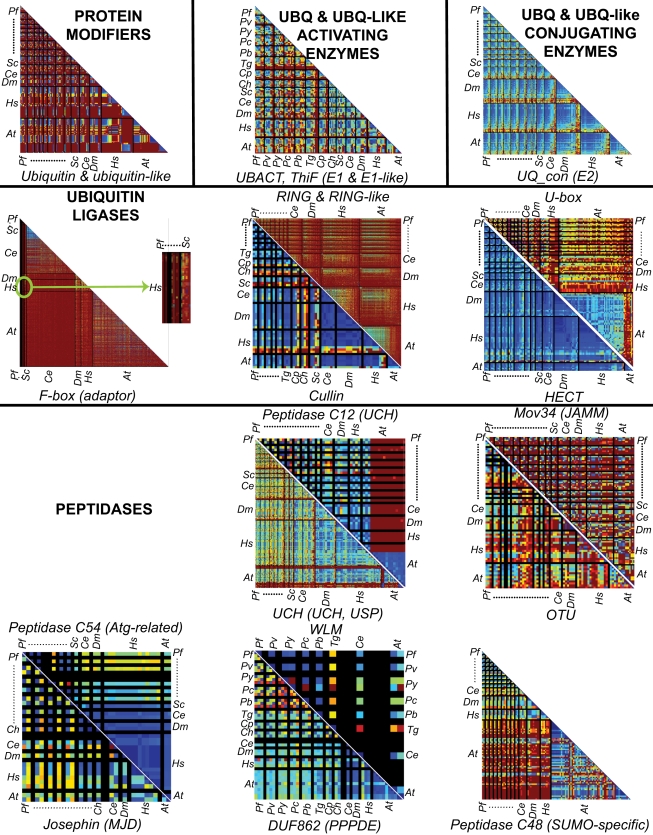
Color matrix representation of by-domain diversity for the 13 proteomes. For each domain, BLASTALL (BLASTP) was run with data from the 13 genomes. Normalized bit scores were plotted following a color scale ranging from “0 = red = very different” to “1 = blue = identical”. All matrices are triangular. Black lines delimit species, with their respective initials are written on each side of the matrix. For each matrix, the order of the species is the following (from left to right or top to bottom): *P. falciparum* (*Pf*), *P. vivax* (*Pv*), *P. yoelii* (*Py*), *P. chabaudi* (*Pc*), *P. berghei* (*Pb*), *T. gondii* (*Tg*), *C. parvum* (*Cp*), *C. hominis* (*Ch*), *S. cerevisiae* (*Sc*), *C. elegans* (*Ce*), *D. melanogaster* (*Dm*), *H. sapiens* (*Hs*), *A. thaliana* (*At*). When the space did not allow writing initials for all species, the first and the last in the succession were indicated separated by dots.

To investigate the global degree of conservation of the predicted UPS proteins, an all-against-all blast search was performed for each domain studied and between the 13 genomes analyzed. The bit scores obtained were reported as a color scale (from red, “highly divergent”; to blue “highly conserved”) in triangular distance matrices. Results of this analysis are shown in figure 2. Using this methodology it is particularly straightforward to realize that ubiquitin/ubiquitin-like activating enzymes and ubiquitin-conjugating enzymes are conserved in all eukaryotic cells including apicomplexan parasites. HECT-ubiquitin ligases and Cullin-ubiquitin ligases are also well-conserved, while RING and RING-like ubiquitin ligases, U-box ubiquitin ligases and ubiquitin ligase adaptors F-box show more diversity. This is particularly striking with regards to RING/RING-like ubiquitin ligases and F-box adaptor proteins where almost every protein considered in our study is divergent from all others, with the exception of U-box, RING/RING-like-containing proteins from *A. thaliana*. In this case, diversity intra-species is much lower than in any other species. The biological significance of this observation remains to be elucidated.

With regards to the eight subclasses of DUBs analyzed, the Josephins (MJD), UCHs (peptidase_C12), autophagins (peptidase_C54) and deSUMOylases (peptidase_C48) families are well conserved within and between species with the exception of a clear differential expansion in the *A. thaliana* UCHs and deSUMOylases. In the other subclasses, relative divergences exist within and between species. An increased divergence can be observed in the metalloprotease (JAMM/Mov 34). The function of the WLM family, usually found only in plant and fungus, in *Plasmodium* and *Toxoplasma* thus deserves to be fully investigated.

**Figure 3:**
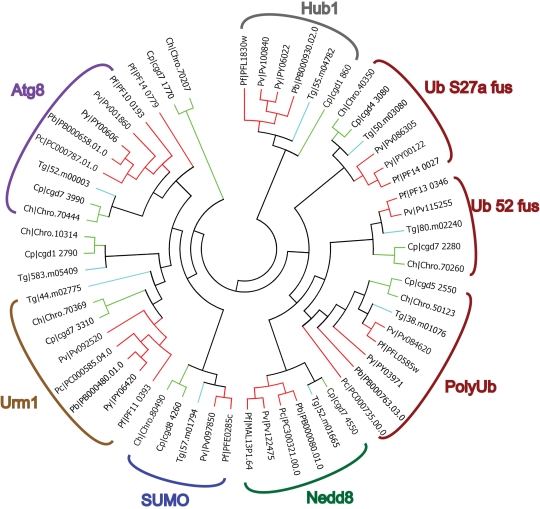
Dendrogram tree of ubiquitin and ubiquitin-like modifiers in *Plasmodium spp.*, *Cryptosporidium spp.* and *T. gondii*.

**Figure 4:**
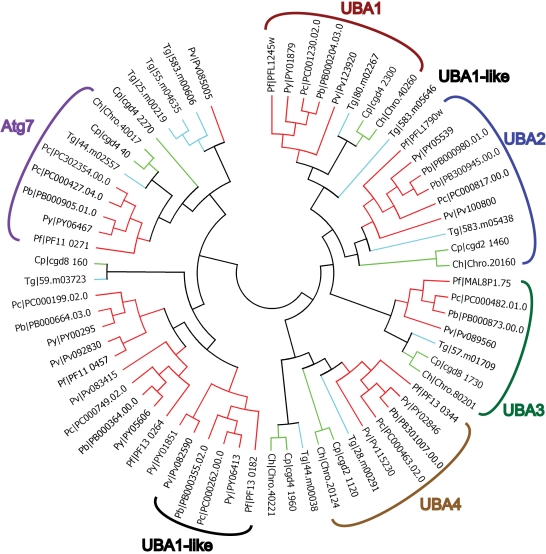
Dendrogram tree of ubiquitin and ubiquitin-like activating enzymes in *Plasmodium spp.*, *Cryptosporidium spp.* and *T. gondii*.

**Figure 5:**
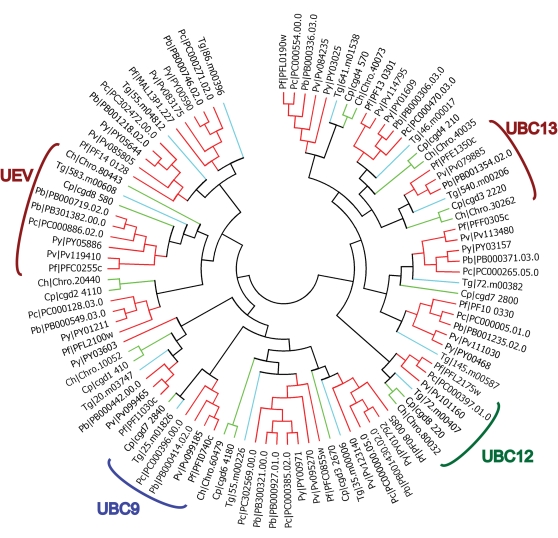
Dendrogram tree of ubiquitin and ubiquitin-like conjugating enzymes in *Plasmodium spp.*, *Cryptosporidium spp.* and *T. gondii*.

**Figure 6:**
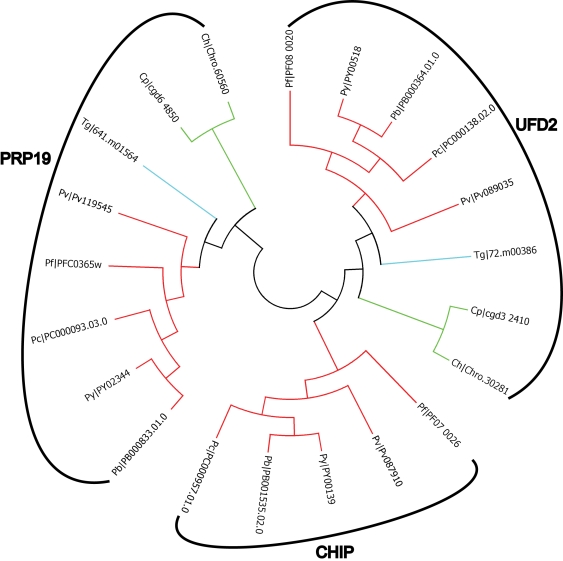
Dendrogram tree of ubiquitin and ubiquitin-like conjugating enzymes in *Plasmodium spp.*, *Cryptosporidium spp.* and *T. gondii*.

When possible, the complete dataset was used to predict apicomplexan functional homologs of known UPS components from data available from the five model organisms investigated here. For each domain, dendrogram trees were built with all the 13 species that we used in this study. For purpose of clarity, only apicomplexan data are presented here. However, our complete results are available for download on the laboratory website (http://lerochlab.ucr.edu/UPS_prediction_data). Figure 3, 4, 5 and 6 show the apicomplexan data for ubiquitin/UBLps, E1, E2, and E3-Ubox enzymes respectively. The rest of the apicomplexan data for the other domains are given in supplemental figure S1.

### Ubiquitin and ubiquitin-like proteins

Ubiquitin is a 76 amino acid protein extensively conserved between all eukaryotic sequences, with similarities in excess of 98% between humans, yeast and apicomplexan parasites [43], [44]. UBLPs generally bear little primary sequence identity to ubiquitin (figure 2), however they share two principle features. First is a compact tertiary structural motif consisting of five beta-sheets and a single alpha helix, termed the ubiquitin fold, in addition to a low complexity C-terminus available for activation and conjugation. Second is a shared biological function through activation, conjugation and reversible modification of a target protein's activity. Since first identification of the first UBLp (the interferon stimulated gene 15, ISG15) in 1979 additional UBLps (at least 10 to date) have been described with an escalating frequency and evidence of UBLps ever-widening role in the modification of cellular processes [17], [23]. Paralogs for polyubiquitin, two ubiquitin-ribosomal protein fusions (Ub-S27a and Ub-52), neural precursor cell expressed developmentally and down-regulated 8 (NEDD8), small ubiquitin-related modifier (SUMO), homologous to ubiquitin 1 (HUB1), ubiquitin-related modifier 1 (URM1) and autophagy 8 (ATG8) have been described in most of the apicomplexans investigated here (figure 3) [43], [44]. The fact that there are missing paralogs more likely reflects the quality of the genome sequence available for the different apicomplexan species (depending upon the status and fold-coverage of their genome projects) rather than absolute absence from the genome. For example, several incomplete sequences from the murine malarial parasites (*P. chabaudi* and *P. yoelii*) contain partial ubiquitin sequences; however it was impossible to definitively assign the final ubiquitin gene based on the sequence available. In addition to ubiquitin/UBLps, several genes were identified by the HMM search that contain the highly related ubiquitin-binding domain (UBD). These typically N-terminal located domains are found in proteins that have evolved to adopt the ubiquitin domain in a non-conjugated role in processes such as signal transduction and proteasomal delivery [45]. These proteins (including paralogs of yeast RAD23 and DSK2) were manually edited from the list of ubiquitin/UBLps for all the organisms investigated.

As in all eukaryotes, ubiquitin is encoded by one of three types of fusion-protein precursors in the apicomplexans investigated here (figure 3). Although multiple copies of these genes may exist in higher eukaryotes, only single copies were identified in apicomplexans. The first type, polyubiquitin, consists of three to five direct repeats of the ubiquitin coding sequence. The two remaining ubiquitin-fusion genes encode N-terminal ubiquitin fused to one of two ribosomal proteins (S27a and S52). In all cases, subsequent proteolytic cleavage of the polypeptide encoded releases the ubiquitin monomers. A suitable steady state level of available ubiquitin monomers is provided by *de novo* synthesis of ubiquitin and recycling of ubiquitin following cleavage from their target proteins. During periods of stress, elevated demands for ubiquitin are met, in part, by increased levels of polyubiquitin expression [46]. Expression data available for *P. falciparum* indicates all three ubiquitin genes are expressed throughout the parasite's life cycle [7], [9], while polyubiquitin also appears to be induced during a heat-shock response [44], [47].

Reversible modification by SUMO is generally associated with processes involving nuclear integrity and function, and more specifically with nuclear transport, subnuclear targeting and genome stability (for review see [48]). More recently, conjugation by SUMO has been suggested to play an additional role of antagonizing the effect of ubiquitin conjugation–an evolution that appears to suggest a complex interplay of protein modification above that of simply activating and inactivating a protein [49], [50]. Although not identified in all *Plasmodium spp*., single copies of genes encoding SUMO have been identified across all the apicomplexan organisms investigated here (figure 3). While higher eukaryotes typically have three to four variants of SUMO, like most single-celled eukaryotes apicomplexans only have one SUMO variant. These appear most similar to SUMO-1 in that they lack an intrinsic sumoylation motif within the N-terminus (ψKXE, where ψ represents a hydrophobic residue and K the targeted lysine), suggesting that polysumoylation does not have a functional role in apicomplexans. Thus, only ubiquitin, by virtue of multiple internal modifiable lysines is capable of forming conjugated polymeric chains on target proteins.

NEDD8, also termed related to ubiquitin 1 (RUB1), is most similar to ubiquitin at the primary sequence level. NEDD8 typically accumulates in the nucleus where its only known target, cullin, is found [51]. As described later, cullins form the scaffold for the SCF (Skp-Cul1-F-box) E3 ubiquitin ligase complexes [52]. NEDD8 appears to play an essential role in cell cycle control in actively proliferating cells and is down-regulated during cell differentiation [53]. Single genes encoding NEDD8 were identified in all apicomplexan families investigated here (figure 3), branching closely with all the ubiquitin-fusion genes as would be expected given the higher primary sequence similarity of this UBL modifier.

Single copies of genes encoding the less characterized UBLps URM1 and HUB1 are found throughout the apicomplexan lineages investigated here (figure 3). Both UBLps have only been recently discovered [54], [55], and little is known about their biological roles. HUB1 is noteworthy for the absence of the typical di-glycine C-terminal motif common to most UBLps, rather having a di-tyrosine motif. As yet, E1 and E2 proteins that would activate and conjugate HUB1 have not been characterized and recent reports suggest that a more “hormonal” role may exist in higher eukaryotes [56]. However, in *S. cerevisiae*, conjugation to proteins involved in mRNA and pre-mRNA splicing have been described, and may more likely reflect the role of HUB 1 in apicomplexans [55]. The second UBLP, URM1, shares very little homology to ubiquitin, but appears more closely related to the *Escherichia coli* sulphur carring proteins ThiS and MoaD involved in thiamin and molybdopterin synthesis, respectively [54]. In *S. cerevisiae*, URM1 has only been found to conjugate to alkyl hydroperoxide reductase 1 (AHP1), suggesting some role in adaptation to oxidative stress may similarly operate in apicomplexans [57].

The autophagy system facilitates degradation of the cytoplasm following engulfment in a vesicle followed by fusion to lysosomes, a process necessary for both cell differentiation and response to starvation. Analysis of mutations in autophagy in *S. cerevisiae* identified two UBLps involved in this system, termed ATG8 and ATG12 [58]. Previous analysis of several apicomplexan and kinetoplast genomes highlighted that while a gene encoding ATG8 could be readily identified across a range of protozoa [43], no evidence exists for the gene encoding ATG12 (see figure 3). ATG12 plays a key role in the initial formation of the autophagosome, while ATG8 is conjugated to the amide group of phosphatidylethanolamine in the membrane, altering the membrane dynamics; thus, ATG8 is unique amongst UBLps in not conjugating a protein. Interestingly, while ATG12 has not been found in kinetoplastids, autophagy has been demonstrated to be active in *Leishmania spp.* and play a key role in parasite virulence [59]. The Pfam search described here identified a single gene in *P. falciparum* as being an ATG12 paralog (table 1, PF14_0779). However, though the predicted polypeptide shares some primary sequence homology to ATG12 from *C. elegans*, it lacks a C-terminal glycine. Further, the cognate E2 and target proteins for ATG12, ATG10 and ATG5, respectively, are absent from *P. falciparum* (as well as the other apicomplexans investigated).

A number of UBLps typical of higher eukaryotes (ISG15, FAT10, UFM1, FUB1) have not been found in this analysis, nor that previously described by Ponder and Bogyo (2007). Although some UBLps may not be expected based on their predicted roles in immune system regulation in higher eukaryotes, their absence, coupled with that of SUMO variants and ATG12 in apicomplexans suggest a more restricted role for UBLps in apicomplexan cell biology. However, analysis of gene expression data (microarray and proteomics) for SUMO, NEDD8, HUB1, URM1 and ATG8, where available (particularly for *P. falciparum* and *T. gondii*), suggests that these UBLps are expressed at all the life stages investigated. These data suggest that ubiquitin/UBLps are essential components in controlling cellular processes throughout apicomplexans complex parasitic life cycles.

### Ubiquitin/UBL activating enzymes (E1)

The first step in the ubiquitin/UBLps activation and conjugation cascade is mediated *via* E1 proteins. A number of isoforms of E1 exist, each responsible for the activation of different ubiquitin/UBLps (for review see [60]). All E1s, however, share a common mechanism of action. The initial step is the ATP-dependent adenylation of the C-terminus of the cognate ubiquitin/UBLp, which is then held in a non-covalent interaction until subsequent attack by an active site cysteine resulting in covalent attachment of the ubiquitin/UBLp *via* a thioester bond. The final step in the mechanism is the transfer of the activated ubiquitin/UBLp to E2 *via* a transesterification reaction.

E1 proteins are characterized by the presence of the ubiquitin activating (UBA) Pfam domain. Additional motifs in E1 are responsible for the correct selection of ubiquitin/UBLp for activation and subsequent E2 to which transfer the activated ubiquitin/UBLp [61]. Whereas the E1 responsible for ubiquitin activation (homologous to UBA1 of *S. cerevisiae*) can deliver activated ubiquitin to several E2 isoforms, the E1s responsible for activating the UBLps SUMO and NEDD8, termed UBA2 and UBA3, respectively, only transfer to a single cognate E2 (see below). UBA1 has two UBA domains on a single polypeptide. UBA2 and UBA3, each only have one UBA domain containing the active site cysteine required for the covalent attachment of activated SUMO and NEDD8, and actually represent one part of a E1 heterodimer complex with AOS1 or APPBp1, respectively [60]. Both AOS1 and APPBp1 each contain one UBA domain, thus resulting in an E1 complex with two UBA domains. The analysis presented here indicates the presence of paralogs for UBA1, UBA2 and UBA3 in all the apicomplexan lineages (figure 4). High level of primary sequence identity in the core of the UBA domains present in UBA1-3 is conserved across all thirteen eukaryotes described in this analysis (figure 2). However, outside of this core homology, sequences diverge rapidly as the functional requirements for these sequences in specifically interacting with different ubiquitin/UBLps and E2 proteins alter. Assigning paralogs for AOS1 and APPBp1 has not been possible here–although unassigned proteins containing UBA domains are present in all apicomplexans investigated here and may well represent functional paralogs for these proteins. Analysis of transcriptional patterns in *P. falciparum* of UBA2 and UBA3 with those of the unassigned UBA containing proteins, as well as searching for existing characterized yeast two hybrid interactions, did not provide any additional clues in defining AOS1 or APPBp1 paralogs [62].

In addition to those described above, three additional E1 proteins are indicated in figure 4. The first, UBA4, is responsible for activation of URM1. Interestingly, UBA4 has only a single UBA motif, but does have a rhodanese homology domain (RHD) [63]. Rhodanese and RHD containing enzymes are responsible for sulphur transfer reactions and form a persulphide bond on their active site cysteines. Interestingly, an E2 for URM1 has not been identified to date, and it is suggested that the RHD may act as a substitute in-built E2 in the transfer of URM1. The second E1, ATG7, is the only E1 that is capable of activating more than one UBL modifier–ATG8 and ATG12 [64], however, as described above, ATG12 does not appear to be present in the protozoa lineage. ATG7 is characterized by a single C-terminal UBA domain with a large N-terminal ATG7 specific motif. The final E1 isoform, termed UBA1-like (due to presence of two UBA motifs), tend to be larger than Uba1. In several eukaryotes, two or more isoforms of ubiquitin E1 exist, however, whether these UBA1-like E1s represent a second ubiquitin E1 or are required for transfer of a different UBL modifier (note no E1 for HUB1 has been assigned should it actually have a conjugating role) remains to be determined.

Proteomic and transcriptomic profiling data available for *P. falciparum* provide extensive evidence for the ubiquitous expression of E1s throughout the parasite's life cycle [7], [9]. Interestingly, detailed analysis of transcription during intraerythrocytic development suggests a temporal pattern of transcript accumulation in the early trophozoite stages when the parasite becomes more metabolically active. Similar data available for *T. gondii* similarly suggest constitutive expression of E1s throughout apicomplexan life cycles.

### Ubiquitin/UBL conjugating enzymes (E2)

Eukaryotes express a number of E2 isoforms, typically of between 17–22kDa (see [65] for review). E2s are readily identified by the presence of a conserved central 150-residue domain that forms a tertiary structure where the cysteine in the active site, which accepts the activated ubiquitin/UBLp from E1 *via* a transesterification reaction, is buried in a shallow groove [66]. The extensive conservation exhibited by the E2 proteins identified in the search of the 13 genomes described here is readily exemplified in figure 2.

The apicompexan parasites investigated here have eight to fourteen E2 proteins, similar to the 14 described for the only other single cell eukaryote *S. cerevisiae* (table 1). The number of E2 isoforms tends to increase with increasing genome complexity. Given the relative completeness of the *Cryptosporidium spp.* genomes, the relative small number of E2 identified in *C. hominis* and *C.parvum*, eight and eleven, respectively, may suggest that these represent a true variation from the mean of 13 to 14 E2s found in *Plasmodium spp.* and *T. gondii*. Specifically, two E2 variant paralogs immediately adjacent to the UEV branch in figure 5 (containing the *P. falciparum* genes PF14_0128 and MAL13P1.227) are only found in *Plasmodium spp.* and *T.gondii* in this analysis and are atypical E2s of up to 54kDa with a long N-terminal extension. N- and C-terminal extensions in E2 are thought to play key roles in recognition and association with E3s and their subsequent protein target and thus these atypical E2s may reflect a specific adaptation in the *Plasmodium* and *Toxoplasma* lineages.

Different isoforms of E2 have distinct roles in regulating downstream functions through specific interaction with distinct E3s ([22] for review and [67]). While several E2s are capable of cascading activated ubiquitin through to different E3s, only single E2 isoforms conjugate to SUMO and NEDD8; UBC9 and UBC12, respectively. Paralogs for both UBC9 and UBC12 are present in all the apicomplexan lineages investigated here (figure 5). One isoform of E2, termed the Ub-E2 variant (UEV), lacks both a key HPN amino acid motif and the active site cysteine in the E2 core and is incapable of conjugating ubiquitin. UEVs instead form a heterodimer with the UBC13 E2 isoform and direct a subset of E3s to conjugate ubiquitin to its target through the side chain of Lys63 (as opposed to more typical conjugation through the side chain of Lys48) [50]. Paralogs for both UBC13 and a UEV are present in all the apicomplexan lineages investigated here (figure 5). Conjugation of ubiquitin through Lys63 generally acts as non-proteolytic signals for processes such as DNA repair [49]. Thus, proteins may be conjugated by polyubiquitin chains, single ubiquitin molecules through more than one lysine side chain and even competitively with SUMO. This diversity of conjugation has important implications in post-translational modifications directing a diverse response in the target protein. Interestingly, strong yeast two-hybrid data in *P. falciparum* indicates a clear association of the UBC13 and UEV paralogs in this organism [62]. One E2 molecule not reported in this analysis is ATG3, which is responsible for conjugation to the UBLp ATG8. This E2 exhibits extreme diversity to that of other E2s and lacks the core E2 Pfam motif used in this analysis. Paralogs exist in all apicomplexans investigated here (PFI0280c, PB000344.03.0, PC000563.02.0, Pv098725, PY04567, chro.80308, cgd8_2650 and 46.m01688).

Extensive gene expression data for nine of the fourteen *P. falciparum* E2s suggest a diverse pattern of steady state mRNA accumulation at different stages of intraerythrocytic development. The fact that different E2 isoforms are expressed at distinct stages in the parasite's life cycle suggests that a temporal profile of delivering ubiquitin/UBLps to different E3s exists, which highlights a potential additional level of temporal control in the UPS system during apicomplexan parasite's life cycles.

### Ubiquitin/UBL ligases (E3)

E3 ubiquitin/UBL ligases are a very diverse group of proteins involved in specifically transferring ubiquitin/UBLps to a given substrate. In all organisms, 48% of the predicted UPS components identified belong to the E3 ubiquitin/UBL ligase family. This high percentage of E3 reflects the specificity that is required for specific substrate recognition. Table 2 summarizes all potential E3 ubiquitin/UBL ligases that have been found in *P. falciparum*, and their homologs in *T. gondii*, *C. parvum*, and yeast. There are three superfamilies of E3 ubiquitin/UBL ligases. HECT ubiquitin ligases have a direct role in catalysis during ubiquitylation, whereas RING (Really Interesting New Gene) finger and U-box E3s are involved in multi-protein complexes. RING finger E3s are the most abundant ubiquitin/UBL ligases.

**Table 2:** Annotated list of E3 ubiquitin and ubiquitin-like ligases in *P. falciparum*, with their homologs in *T. gondii*, *C. parvum* and *S. cerevisiae.*

Domain	*P. falciparum*	*T. gondii*	*C. parvum*	*S. cerevisiae*	Annotation
HECT	MAL8P1.23	86.m00385	none	TOM1	GO: mRNA transport; similar to UPL1/UPL2 in *A. thaliana*
HECT	PF11_0201	64.m00324	cgd8_1200	HUL5	similar to UPL6/UPL7 in *A. thaliana*
			cgd1_1920		
HECT	MAL7P1.19	80.m02344	none	UFD4	cytoplasmic E3 for degradation of ubiquitin fusion protein
HECT	PFF1365c	25.m01837	cgd7_4990	none	GO: cell proliferation; UPL5 in *A. thaliana*
		59.m03523			
		72.m00400			
Cullin	PF08_0094	80.m02207	cgd4_3150	CDC53	structural protein of SCF complexes
Cullin	PFF1445c	none	none	CUL8	possible role in anaphase progression;
U-box	PF08_0020	72.m00386	cgd3_2410	UFD-2	ubiquitin chain assembly factor E4; ubiquitin fusion degradation protein
U-box	PFC0365W	641.m01564	cgd6_4850	PRP19	splicing factor associated with the spliceosome
U-box	PF07_0026	none	none	none	similar to CHIP in *A. thaliana* and *H. sapiens*
RING finger	PFI0470w	none	cgd2_2410	SSM4	Deg1 signal-mediated degradation pathway; GO: mRNA turnover and stability
RING finger	MAL13P1.405	20.m03749	cgd8_4800	none	have homologs in *A. thaliana* only; unknown function
RING finger	PF14_0215	540.m00334	cgd8_2560	none	similar to HRD1-like; GO: ERAD pathway
RING finger	PFC0510w	50.m05636	cgd7_4170	HRD1	involved in the ERAD pathway
			cgd1_1790		
RING finger	PFE1490c	46.m00026	cgd8_3470	none	similarities with RIE1 in *A. thaliana* (seed development)
RING finger	PF10_0276	42.m00120	cgd7_4910	none	unknown function
		44.m02707			similar to ATL4 in *A. thaliana*
RING finger	PFF0355c	74.m00769	none	none	unknown function; found in Apicomplexa only
	PF14_0054				
RING finger	PF10_0072	80.m03951	cgd1_1950	none	unknown function
RING finger	PFF0755c	57.m01707	cgd2_2950	RKR1	GO: chromatine structure
RING finger	PFC0740c	57.m01858	cgd4_1360	none	unknown function; GO: cell growth regulation
			cgd3_1260		
RING finger	PFL0440c	540.m00204	cgd5_3990	ASI1	with ASI2 and ASI3 ensures the fidelity of SPS-sensor signalling
			cgd5_3970		
RING finger	PFE100w	none	cgd5_3900	PEX2	component of the CORVET complex
	PFI0805w				
RING finger	PFL1620w	none	none	DMA1	spindle position and orientation
				DMA2	
RING finger	PFC0175w	none	none	YKR017C	homologous to ariadne ubiquitin conjugating enzyme binding protein in *H. sapiens*
RING finger	PFF1325c	20.m03922	cgd2_1820	none	unknown function
RING finger	PFC0610c	583.m00699	cgd4_4310	PIB1	GO: endosomal trafficking, vacuolar trafficking
RING finger	PFF0165c	35.m01589	cgd2_880	BRE1	involved in histone H2B ubiquitination
RING finger	MAL7P1.155	none	none	none	unknown function; possible cytoskeleton-related
RING finger	PF14_0416	42.m00073	cgd6_3300	CWC24	element of the spliceosome
RING finger	PF14_0139	50.m03082	cgd8_3720	none	GO: cell proliferation
RING finger	PFL1705w	49.m03145	cgd7_4960	YER068W	Not-like; component of the CCR4-Not complex
RING finger	PFC0425w	none	none	none	unknown function; found in Apicomplexa only
	MAL13P1.224				
RING finger	PFL0275w	20.m03824	cgd7_3320	none	possible topoisomerase 1
RING finger	PFD0765w	none	none	none	unknown function
RING finger	PF11_0244	20.m03803	none	none	unknown function; found in Apicomplexa only
RING finger	PF10_0046	76.m01590	cgd3_2060	none	similar to CIP8 in *A. thaliana*
RING finger	PFF1180w	none	cgd1_2640	APC11	element of the anaphase promoting complex/cyclosome
RING finger	PFC0845c	none	cgd8_930	RBX1	element of the Skp1-Cullin-Fbox complex
RING finger	PFB0440c	583.m05584	cgd3_3460	none	unknown function
RING finger	MAL13P1.216	641.m01484	none	RAD5	component of the SWI/SNF pathway
RING finger	PFL2440w	42.m00128	cgd4_140	RAD16	component of the SWI/SNF pathway
RING finger	PF10_0117	none	cgd2_1750	none	unknown function
RING finger	PFE0610c	641.m02557	cgd1_3300	TFB3	component of the nucleotide excision repair pathway
RING finger	PF13_0188	none	cgd5_1200	none	unknown function; found in Apicomplexa only
RING finger	MAL13P1.122	none	cgd5_400	none	unknown function; found in Apicomplexa only
RING finger	PFC0690c	none	cgd7_1170	YDR266C	role in partionning of cytoplasm
RING finger	PF11_0330	59.m03727	none	none	possible SUMO ligase

Our search identified four HECT domain-containing proteins in *P. falciparum*, and other apicomplexans. Three of them have a homolog in *S. cerevisiae*: TOM1, UFD4, and HUL5 (HUL5 has unknown functions). The fourth HECT-domain protein that we identified in apicomplexans does not match any protein from yeast but is similar to UPL5 in *A. thaliana* (see table 2). UPL5 has an unknown function, but is annotated as potentially involved in cell proliferation. TOM1 (MAL8P1.23 in *P. falciparum*, and 86.m00385 in *T. gondii*) has been recently described as being involved in cell cycle arrest after DNA damage, mediating CDC6 ubiquitylation, a protein essential to initiation of DNA replication [68]. UFD4 (MAL7P1.19 in *P. falciparum*, and 80.m02344 in *T. gondii*) is involved in the ubiquitin fusion degradation pathway (UFD pathway) [69], which results in polyubiquitylation of ubiquitin fusion proteins that do not fall into the N-end rule pathway (the N-end rule relates the *in vivo* half-life of a protein to the identity of its N-terminal residue; for example, in eukaryotes, a protein with an isoleucine at its N-terminal end will be targeted to the proteasome more rapidly than a protein with a glycine, which is a stabilizing residue; see [70] for a review). Little is known about the UFD pathway, and its physiological functions remain unknown. Recent works indicate that UFD4 is involved in controlling the degradation of RAD4, a nucleotide excision repair protein [71].

U-box proteins are another family of ubiquitin ligases that are structurally similar to RING finger proteins but lack the metal binding sites (see [26] for a review). A sub-group of U-box proteins is also termed E4 ubiquitin chain assembly factor, and is known for its ability to add a polyubiquitin chain on a substrate already primed for degradation by oligoubiquitylation [72]. We identified two U-box domain-containing proteins that are present in all apicomplexan parasites: the *S. cerevisiae* UFD2 (PF08_0020 in *P. falciparum*, and 72.m00386 in *T. gondii*) and PRP19 (PFC0365w in *P. falciparum*, and 641.m01564 in *T. gondii*) homologs (figure 6). UFD2 is an E4 ubiquitin ligase, involved in the UFD pathway [72]. In yeast, UFD2 interacts with the AAA ATPase (ATPase associated with various activities) CDC48, which possesses a chaperone-like activity and mediates ubiquitin-dependant endoplasmic reticulum associated degradation (ERAD) pathway [73]. Richly *et al.* [74] proposed a short ubiquitylation chain-dependant escort pathway to the proteasome that would involve UFD2, which adds ubiquitin proteins to a substrate already mono- or di-ubiquitinated. In this escort pathway, the Cdc48-Npl4-Ufd1 would act to restrict chain length to four to six ubiquitin. CDC48 (p97 in mammalians) is involved in several different functions in cell, such as retrotranslocation from the ER to the cytosol (quality control), transcriptional control or cell cycle regulation (see [75] for a review). CDC48 is the cyclin-dependant kinase (CDK) that promotes cell cycle progression in yeast. Previous works indicate that CDC48 regulates the stability of several cell-cycle regulators in a UPS-dependant manner. CDC48 was also found to regulate the stability of proteins involved in controlling the expression of genes involved in fatty acid metabolism in yeast (see [75] for a review). We used BLASTP to identify homologs of proteins involved in the escort pathway and do not carry UPS-related domains (and are thus absent from our dataset). We predicted CDC48, Ufd1 and Npl4 in *P. falciparum*, PFF0940c, PF14_0178 and PFE0380c respectively (see supplemental Table S2). All these proteins seem to be well conserved in *P. falciparum*. Furthermore, clear orthologs of CDC48, Ufd1 and Npl4 are predicted in *P. yoelii*, *C. parvum*, *C. hominis*, and *T. gondii* (from OrthoMCL-DB, [76], see supplemental Table S2). These findings could indicate that the Ufd2-dependant Cdc48-Npl4-Ufd1 escort pathway exist in *P. falciparum* and other Apicomplexa, with functions similar to the ones observed in yeast.

PRP19 is an oligomeric U-box-containing E3 ligase [77] that plays a role in mRNA splicing [78], spliceosome activation and recycling [79], [80], and DNA damage response [81]. PRP19 is part of a complex consisting of at least eight units, of which CDC5 and PLRG1 (Pleiotropic regulator 1) [78]. Lu and Legerski [81] demonstrated that, in DNA damage conditions, PRP19 is ubiquitinated. The authors showed that ubiquitinated PRP19 fails to interact with either CDC5 or PLRG1, and over expression of PRP19 reduces the levels of apoptosis after exposure of cells to DNA damage. Little is known about apoptosis or programmed cell death in Apicomplexa. Apoptosis-like events have been described in *P. falciparum* and *P. berghei* [82], [83]. Al-Olayan and co-workers suggested that apoptosis could be a possible mechanism for limiting intensity of infection in the mosquito by *P. berghei* (see [84] for review). Unfortunately no data is currently available about the function or the expression pattern of PRP19 homolog (PFC0365w) in *P. falciparum* ookinetes. In *T. gondii*, the homolog of PRP19 (641.m01564) contains WD40 repeats, which are known to be involved in a wide array of cellular processes ranging from signal transduction and transcription regulation to cell cycle control and apoptosis [85], [86]. However, the functions of PFC0365w and 61.m01564 remain to be elucidated.

A third U-box containing ubiquitin ligase, that is absent in yeast, has been identified in *Plasmodium* species (PF07_0026 in *P. falciparum*) and shares extensive homology with the human protein CHIP (C-terminal of Hsp70-interacting protein). Like UFD2, CHIP has been described as being an E4 ubiquitin ligase in human. A particular feature of CHIP is that its catalytic activity requires its homodimerization through the U-box domain (see [87] for review). CHIP is known to be involved in protein quality control by promoting ubiquitylation of denatured proteins in an Hsp70/Hsp90-dependant manner. CHIP is also involved in heat shock response and prevention of apoptosis. However, the physiological substrates of CHIP still remain unidentified. Previous experiments suggested that CHIP might have multiple functions that could be proteolytic either dependent or independent from proteasome degradation [88]. However, the physiological role(s) of CHIP remain(s) unknown. In *P. falciparum*, the CHIP homolog PF07_0026 is mainly expressed at the sporozoite stage (mosquito stage). Interestingly we also identified CHIP homologs in *P. berghei*, *P. chabaudi*, *P. vivax* and *P. yoelii* (respectively PB001535.02.0, PC000957.01.0, Pv087910, PY00139) whereas no homolog was found in yeast *Cryptosporidium spp*. and *T. gondii*. Thus, it can be hypothesized that CHIP homologs in *Plasmodium* are involved in the hepatic infection stage, although a precise role remains to be determined.

Cullin-containing proteins belong to the E3 ubiquitin ligase family. In association with a RING finger E3 and a substrate recognition protein such as F-box proteins, they form Cullin-RING-Ligases (CRLs). The most famous CRLs are the Skp1-Cullin1-Fbox (SCF) complex, containing the cullin protein CDC53 in yeast, and the Anaphase Promoting Complex/Cyclosome (APC/C), containing the cullin protein APC2 in yeast. Both the SCF and the APC/C are involved in cell-cycle progression (see [89] for review). A homolog to CDC53 has been found in each apicomplexan (PF08_0094 in *P. falciparum*, and 80.m02207 in *T. gondii*). The SCF contains at least four subunits: CDC53 (Cullin1) which is stabilized by the ubiquitin-like modifier NEDD8, the RING E3 RBX1, the adaptor protein Skp1 and a F-box protein for substrate recognition (see [39] for review). The present study allowed us to confirm the presence of F-box proteins as well as NEDD8 and RBX1 homologs in apicomplexan genomes (table 1) (MAL13P1.64 and PFC0845c respectively in *P. falciparum*, see figure 3). Using BLASTP, we identified homologs of Skp1 in the genomes of each apicomplexan (MAL13P1.337 in *P. falciparum*, see supplemental table S3). The minimum components that are required for the cell-cycle regulator SCF are therefore all present in apicomplexan genomes. The situation is different as far as the APC/C is concerned. While a homolog to CUL8 and the RING finger APC11 involved in the APC/C were recognized by our analysis (respectively PFF1445c and PFF1180w in *P. falciparum*), the cullin classically involved in the APC/C (APC2) was not found in apicomplexan parasites. The role of CUL8 is not well known. Previous data suggest that it may be involved in anaphase progression [90] in a CRL other than the classic APC/C. The presence of a slightly different APC/C in *P. falciparum* is not entirely unexpected. Cell division during schizogony is apparently asynchronous in *P. falciparum*, that is, several rounds of DNA replication/DNA division occur before final cytokinesis, instead of the paradigm of successive cycles of alternating DNA replication/DNA division/cytokinesis ([91]–[93]).

RING finger and RING-like E3 ubiquitin ligases are the largest group of E3s. They do not have a direct catalytic role in linking ubiquitin and ubiquitin-like modifiers. They rather act as adaptor partners: RING E3s interact with both an E2 ubiquitin-conjugating enzyme (that is carrying ubiquitin) and its given specific substrate, bringing the substrate in close proximity with ubiquitin. Since RING and RING-like E3s are involved in substrate recognition, and given the diversity of proteins that are targeted by the UPS, a large diversity of RING and RING-like E3s is expected (homologs of RBX1 and APC11 are only two of the numerous RING and RING-like proteins, up to 55 in *T. gondii*, that were functionally identified by our analysis). For example, we have found potential RAD16, RAD5, and TFB3 homologs (PFL2440w, MAL13P1.216, and PFE0610c repectively in *P. falciparum*, and 42.m00128, 641.m01484, and 641.m02557 repectively in *T. gondii*), which are known to be involved in nucleotide excision repair (NER) [94]. Previous work showed that RAD16 mediates histone H3 acetylation before global nucleotide excision repair [95]. Interestingly, several of the RING E3s that were found in apicomplexan parasites are potentially involved in mRNA turnover and stability (PFI0470w in *P. falciparum*) or pre-mRNA maturation (PF14_0139 and PF14_0416 in *P. falciparum*, and 50.m03082 and 42.m00073 in *T. gondii*) [96]. Consistent with previous work, this observation suggests that regulation of mRNA stability is a major mechanism of gene regulation in *P. falciparum* [10]. Several others of the identified RING E3s are potentially involved in histone ubiquitination (*e.g.* PfBre1 PFF0165c in *P. falciparum*, 35.m01589 in *T. gondii*), or chromatin remodeling and silencing (PF10_0046 in *P. falciparum* and 76.m01590 in *T. gondii*). Recent data have increasing shown that epigenetic mechanisms play a key role in the control of gene expression in Apicomplexa. In *Plasmodium*, chromatin modeling is one of the proposed mechanisms that control allelic exclusion of the *var* virulence genes [97], [98]. In *T. gondii*, acetylation of histone H4 or acetylation on lysine 9 of histone H3 as well as trimethylation of lysine 4 of histone H3 were shown to be located close to the 5′ UTR of the active genes [99]. Increasingly, evidence suggests that the precepts of the universal “histone code” and the molecular mechanisms that underpin reversible histone modification similarly applies to Apicomplexa, although much yet remains to be elucidated.

It is interesting to notice that several RING-domain proteins found in apicomplexans do not cluster with any other known protein. In *P. falciparum*, PF14_0054 and PF13_0188 are two examples of proteins with no evident homologs in the organisms analyzed, besides their apicomplexan counterparts. With regards to their expression profiles [7], [9], PF14_0054 is expressed at the sporozoite and the late schizont stages of *Plasmodium*'s life cycle, which suggests that this protein may be specific to apicomplexan processes such as parasite invasion. However, functions of several of the predicted parasitic RING and RING-like proteins remain to be elucidated, either because there are no known homologs in other model organisms, or because the function of the matching homolog remain unknown.

**Figure 7:**
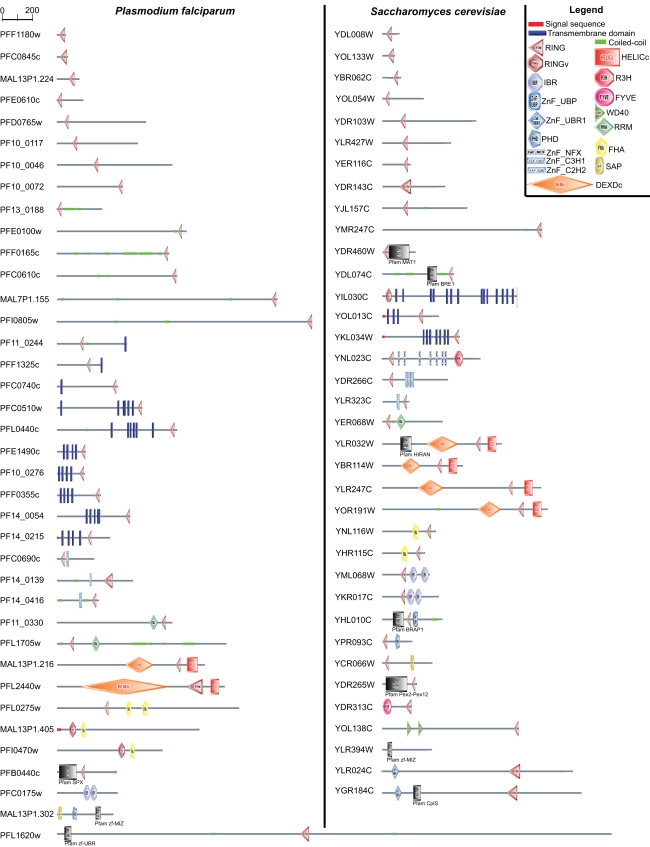
Domain architecture of RING and RING-like E3 ligases in *P. falciparum* compared to *S. cerevisiae*. Domain architectures were retrieved using batch access to SMART database (http://smart.embl-heidelberg.de/smart/batch.pl). Domains in black and grey were retrieved from the Pfam database (http://pfam.sanger.ac.uk). Transmembrane segments are predicted with the TMHMM2 program, coiled coil regions are predicted with the Coils2 program, and signal peptides are predicted with the SignalP program. Legend: RING = RING finger; RINGv = C4HC3 RING-variant; IBR = in between RING fingers; ZnF_UBP = Ubiquitin Carboxyl-terminal Hydrolase-like zinc finger; ZnF_UBR1 = Putative zinc finger in N-recognin, a recognition component of the N-end rule pathway; PHD = plant homeodomain zinc finger; ZnF_NFX = zinc finger domain repressor of transcription; ZnF_C3H1 = zinc finger domain; ZnF_C2H2 = zinc finger domain; DEXDc = DEAD-like helicases superfamily; HELICc = helicase superfamily c-terminal domain; R3H = Putative single-stranded nucleic acids-binding domain; FYVE = zinc finger present in Fab1, YOTB, Vac1, and EEA1; WD40 = WD40 repeats; RRM = RNA recognition motif; FHA = Forkhead associated domain; SAP = Putative DNA-binding (bihelical) motif predicted to be involved in chromosomal organisation; Pfam MAT1 = CDK-activating kinase assembly factor MAT1; Pfam BRE1 = CDK-activating kinase assembly factor MAT1; Pfam HIRAN = HIP116, Rad5p N-terminal domain, found in the N-terminal regions of the SWI2/SNF2 proteins; Pfam BRAP2 = BRCA1-associated protein 2; Pfam Pex2-Pex12 = Pex2/Pex12 amino terminal region; Pfam ClpS = ATP-dependent Clp protease adaptor protein ClpS; Pfam SPX = SYG1, Pho81 and XPR1 domain; Pfam zf-UBR = Putative zinc finger in N-recognin (UBR box); Pfam zf-MIZ = MIZ/SP-RING zinc finger. The grey bar represents the primary structure of proteins.

Whilst prediction of function based on sequence homology can be misleading, a study of the domain architecture of RING and RING-like E3 ligases may highlight additional features. *P. falciparum*'s RING and RING-like E3 ligases were investigated using SMART to predict all protein motifs present([100], see material and methods). Results are shown in figure 7. This analysis revealed that *P. falciparum* RING and RING-like E3 ligases possess major domain architectures found in E3 ubiquitin/UBL ligases from other model organisms: an N-terminal or C-terminal RING domain, a RING domain associated with a zinc finger domain such as C2H2 domain, the architecture RING/RNA recognition motif (RRM), RING/helicase, RING/forkhead-associated domain (FHA), and RING/in between RING (IBR). It is interesting to observe that predicted *Plasmodium* RING E3s with a single RING domain appear more abundant than those of yeast. Several of these proteins carry several coiled-coil regions, *e.g.* up to six in PFF0165c. Coiled-coil domains are known to be involved in regulation of gene expression and many other biological processes (see [101] for a review). The role of coiled-coil domains in host-pathogen interactions has particularly been studied. The molecular cross talk that occurs between a pathogen and its given host is complex. The pathogen has to collect and process diverse host signals in order to modulate the expression of its virulence genes. In gram-negative bacteria, coiled-coil proteins are involved in type III secretion systems that are used to deliver virulence effector proteins into, or close to, the host cell (see [102] for a review). In *Agrobacterium tumefaciens*, the histidine kinase VirA activates the expression of virulence genes in response to multiple wound-derived plant signals, *via* the involvement of coiled-coil structures [103]. The HIV protein gp41 contains a conserved coiled-coil domain that is critical for the entry of the virus into the host cell [104]. Thus, the apparent abundance of coiled-coil-containing RING E3 ligases in *P. falciparum* may provide a link to the particular virulence of this parasite and would appear to warrant further investigation.

RING E3 ubiquitin ligases with one or multiple predicted transmembrane domains are also more abundant in *P. falciparum* than in yeast (ten and three proteins, respectively). Membrane bound E3 ubiquitin ligases have been shown implicated in the regulation of immune recognition during virus infections (see [105] for a review). The abundance of such RING E3 ubiquitin ligases would be of obvious interest in understanding how chronic infections are established during infection by these parasites.

### DUBs and DUBLs enzymes

DUBs are responsible for generating ubiquitin precursors from fusion protein and polyubiquitin chains. They are involved in the disassembly of polyubiquitin chains for the maintenance of the available ubiquitin within the cell. DUBs are also responsible for the editing of ubiquitin conjugates to alter their commitment for proteolysis [28], [106]. In these roles, DUBs have been shown to be associated with proteasome subunits [107], [108]. A second important pathway associated with deubiquitylation is the control and degradation of membrane protein trafficking to the vacuole/lysosome [109]–[112]. Furthermore, DUBLs have been shown to play additional key roles in eukaryotic cells. For example, the SUMO deconjugating enzymes (also termed SENPs) play a role in the control of transcription [113], the autophagy-related DUBLs similarly regulate vacuole targeting pathways [114], [115] and the WLM family is thought to be specifically involved in desumoylation and chromatin structure maintenance [29]. Increasing data indicate that most DUBs/DUBLs regulate a limited number of specific substrates, where they interact either directly with ubiquitin/UBLp, or with the target protein to which ubiquitin/UBLp is conjugated.

Between 20 to 40 DUBs and DUBLs were found in apicomplexan genomes (table 1), while 165 were detected in *A. thaliana*. Moreover, most of the major DUBs and DUBLs subfamilies were found within each of the apicomplexan genomes. The few exceptions in *P. chabaudi* and *P. berghei* are probably the consequences of weak genomic sequences coverage for these two apicomplexan species.

UCH-L3, which was identified in all Apicomplexa, is known to cleave ubiquitin and the UBLp NEDD8. The first active DUBs (UCH-L3 and UCH-L5) were recently identified in *P. falciparum* and *T. gondii*, and showed to both react with ubiquitin and NEDD8 [116], [117]. SUMOylases (SENPs), of which at least two are present in apicomplexan genomes, are known to be SUMO-specific proteases but have also been shown in some cases to cleave NEDD8. UBLps, such as ATG8 involved in autophagy, are also processed by distinct proteases, such as the Autophagin peptidase C54, of which one isoform has been identified in all Apicomplexa investigated here.

Determination of target specificity *in vivo* has been challenging in other model organisms. It is possible that *in vivo* protein localization and the presence of adaptors such as E3 ligases can increase specific interactions [118]. Despite the challenge of target identification, the function of several DUBs have been clearly identified and implicated in critical cellular process. The fact that all DUBs subclasses are present in apicomplexans suggests that several of their functions are conserved. For example, deubiquitination of proteins at the proteasome lid is necessary for protein degradation and recycling of ubiquitin. Various DUBs, such as the metalloprotease JAMM (RPN11 and RPN8 in yeast) the UBP6 in yeast or UCH-L5 in mammalian, have been found in complex with proteasome subunits.

Increasing evidence suggests that several DUBs are implicated in remodeling chromatin structure, transcriptional regulation and gene silencing. In yeast, UBP8 and UBP10 have been implicated in the dynamic histone monoubiquitination of H2B. The presence of UBP8 correlates with changes in transcriptional regulation [118] whilst UBP10 is required for the recruitment of the silencing factor Sir2 [119]. Both of these DUBs have homologs in Apicomplexa (eg. PFI0225w and PF14_0145 in *P. falciparum*, respectively) and could be potentially involved in the regulation of parasite virulence genes, principally the *var* gene family in *P. falciparum*.

DUBs are also implicated in the endocytic pathway and intracellular traffic [112]. JAMM and USP proteases (AMSH and USP8 in human) as well as the human OTU (VCIP135) protease have all been shown to have a role in endocytosis and vesicle assembly, respectively. Whether their homologs share the same function in trafficking in Apicomplexa warrants further interest. However, recent studies in antimalarial drug resistance have identified a strong genetic association between drug resistance in *Plasmodium* and a locus containing a DUB related to USP7 [120]. Further studies will be required to validate the potential role of DUBs in the evolution of parasite drug resistance.

Expression profiles analysis [7], [9] reveals that all the putative DUBs/DUBLs identified here were expressed in at least one stage of the *P. falciparum* life-cycle, providing additional evidence for a functional role in the parasite's life cycle.

### Conclusion

The present study allowed for the identification of up to 114 proteins that are predicted to be involved in the UPS of *P. falciparum* and other Apicomplexa. All apicomplexans possess the complete machinery that is required to ubiquitylate proteins (*i.e.* ubiquitin and ubiquitin-like modifiers, E1 enzymes, E2 enzymes, E3 enzymes, and deubiquitinases). Ubiquitin and the common UBL modifiers SUMO, NEDD8, HUB1, URM1, and ATG8 were identified in apicomplexans. However, several UBLps are missing, such as SUMO variants and ATG12, although it has been suggested that autophagy is one of the parasite's death pathway [121]. Further investigations are required to elucidate the role of UBLps in apicomplexan biology. Our results also highlighted apicomplexans-specific features in enzymes involved in transferring ubiquitin and UBL modifiers to a target substrate. For example, two E2 variants found only in *P. falciparum* and *T. gondii* have up to triple the molecular weight of E2s found in other organisms, which could reflect adaptation of *Plasmodium* and *Toxoplasma* lineages. Finally, the superfamily of E3 ubiquitin ligases is very diverse, and several of the E3s predicted in apicomplexans do not find homolog in other eukaryotic organisms. Such proteins could have a cellular role directly linked to parasitic processes, such as invasion. The over-representation of RING E3 ligases that contain coiled-coil domains, known to play important role in host-pathogen interactions, supports this hypothesis.

**Figure 8:**
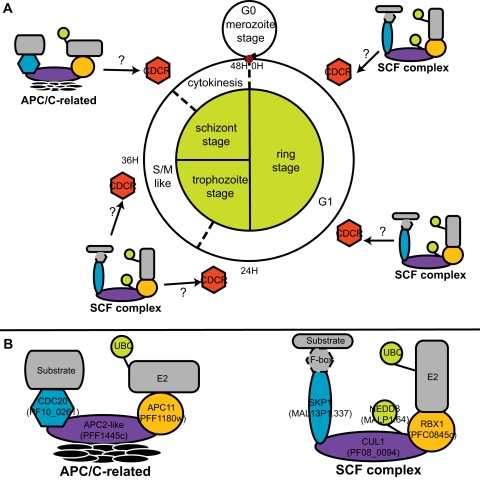
Proposed representation of *Plasmodium falciparum* erythrocytic cell cycle and two predicted regulatory complexes, a Skp1-Cullin-F-box (SCF) complex and an anaphase-promoting complex-related (APC/C-related). A: the 48 hours erythrocytic cell cycle of *P. falciparum*. Morphological stages are given in the inner circle; the outer circle proposes corresponding classical cell cycle phases. H = hours; CDCR = cell division cycle related. The red dot represents when invasion of red blood cells by merozoites occurs. B: detailed APC/C-related and SCF complexes in *P. falciparum*. Identifiers from PlasmoDB are given in parenthesis.

An example of apicomplexan adaptation in the UPS is an apparently modified APC/C (called APC-related). In yeast, SCF and APC are known to play a fundamental role in cell-cycle control. All proteins known to be involved in the SCF machinery are present in the *Plasmodium* genome. The presence of a modified APC complex in the atypical erythrocytic *Plasmodium* cycle is not surprising. The cell cycle in *Plasmodium* can be closely related to the early embryogenesis division observed in *D. melanogaster* with a depletion of key cell cycle regulators. During the first 13 cell divisions of a *D. melanogaster* zygote, the divisions are rapid, synchronous and occur in the absence of cytokinesis and detectable gap phase. These divisions result in the formation of a syncytial cell containing a large number of nuclei in a single cytoplasm. In *Plasmodium*, divisions during schizogony are rapid and asynchronous with up to four or five rounds (four rounds equals 16n and five rounds equals 32n) of DNA synthesis and mitosis during the trophozoite and early schizont stages. Cytokinesis takes place late in the cycle during the mature schizont stage. This is a major divergence from the classical cell-cycle events that consists of a linear succession of G1/S/G2/M phases. A proposed model for the *Plasmodium* cell-cycle control is presented in figure 8 where the classical SCF complex could be involved in the control of the checkpoint between the gap G1 and S phase. The expression profile of the cullin analogue (highly expressed in the trophozoite and schizont stages) potentially involved in the parasite SCF complex reinforces this hypothesis. It will then be expected that the modified APC complex could regulate the atypical parasite cytokinesis. Interestingly, proteasome inhibitors have been shown to block parasite cell-cycle progression at the ring and late schizont stage [122]. These observations help validate the hypothesis of two major parasite cell-cycle checkpoints at the G1/S and the cytokinesis phases.

The number of genes that our work predicted to encode components of the UPS in *Plasmodium* and other apicomplexa is a good estimate when compared to other eukaryotic organisms. Our results demonstrate that E3 ubiquitin/ubl ligases remain one of the most specific components of the UPS. The presence of numerous and diverse E3s, and in particular RING domain-containing proteins, suggests that target-specific ubiquitylation *via* E3 ligases is a complex and important part of cellular regulation in eukaryotic cells, including apicomplexan parasites. In terms of sequence homology, when counterparts exist, most of the ubiquitin ligases identified in Apicomplexa display divergences from their human-host counterpart. The domain architecture analysis for *P. falciparum*'s RING finger E3s revealed an abundance of coiled-coil domain. Since such domains have been shown to play a major role in signal transduction during the molecular cross-talk that occurs during a viral or a bacterial infection, our hypothesis is that E3 ubiquitin/UBL ligases in *P. falciparum* and other Apicomplexa are involved in pathogen virulence and/or pathogenicity.

The potential to treat apicomplexan parasites *via* drugs that target the proteasome component of the UPS has been established in *P. falciparum*. However, this potential to exploit these drugs is limited. The latest generations of drugs that target the UPS have focused more on the specificity of the action of E3 ligases and DUB/DUBLs. Given the apparent apicomplexan diversity in these proteins, opportunities to develop small molecule inhibitors specific against the the apicomplexan-specific E3 ligase and DUB/DUBLs offer some possibility for new, much needed, therapeutics for these devastating global diseases.

## Materials and Methods

### Proteome and domain motifs datasets

Full proteome datasets from the following thirteen organisms were downloaded: *P. falciparum, P. vivax, P. yoelii, P. berghei, P. chabaudi, C. parvum* C. *hominis, Toxoplasma gondii, S. cerevisiae, C. elegans, D. melanogaster, A. thaliana* and *H. sapiens*. Additional information regarding data sources and release information are given in table S4. Twenty-four Pfam motifs, found throughout the UPS, were selected (focusing on ubiquitin and UBLp, E1 and E1-like enzymes, E2 enzymes, E3 and E3-like enzymes and DUB/DUBL enzymes). Their Pfam HMM profiles were downloaded from the Pfam_ls HMM library version 22.0 [123] at http://pfam.janelia.org (Pfam accession numbers: ubiquitin, PF00240; APG12, PF04110; MAP1_LC3, PF02991; UPF0185, PF03671; Urm1, PF09138; UBACT, PF02134; ThiF, PF00899; UQ_con, PF00179; zf-C3HC4, PF00097; zf-RING-like, PF08746; zf-MIZ, PF02891; Cullin, PF00888; U-box, PF04564; F-box, PF00646; HECT, PF00632; Josephin, PF02099; Mov34, PF01398; OTU, PF02338; DUF862, PF05903; WLM, PF08325; Peptidase C12, PF01090; Peptidase C48, PF02902; Peptidase C54, PF03416; UCH, PF00443).

### Protein identification using HMM search

Components of the HMMER 2.3.2 package (released in October 2003) were used throughout the study [124]. This software can be freely downloaded from http://hmmer.janelia.org. The program hmmsearch was used to analyze domain distribution among all proteome datasets and to extract sequences that carry the Pfam domains described above. HMM searches were run using a series of incrementally increasing threshold E-values, from E-value ≤1 to E-value ≤0.1, and results were checked for false positives. Threshold E-value ≤0.5 gave the best quality results, and thus was used in the present study.

### Building colored distance matrices

Proteins carrying same domain motifs were pair aligned by BLASTP. Output bits scores were used as protein distance values: the higher the values are, the more proteins are similar and therefore could be evolutionary closed. All scores below 20 were set to 20. Values were scaled in the range [0,1] by normalizing using a global transformation (d = minimum score/score). A color-mapping matrix, from red to blue, was built, using MATLAB® 7, from the normalized scale. The red color means “highly divergent”, and blue means “highly conserved”.

### Building dendrogram trees

Protein sequences were aligned using ClustalW [125]. For each protein family, bootstrap parameters were set to 10 times the number of proteins in the set; for example, a set containing 100 proteins was boostrapped 1000 times. Dendrogram trees were generated from ClustalW multiple alignments, using the maximum likelihood method in the PHYLIP package [126]. Trees were visualized with MEGA4 [127] and manually annotated.

### Domain architecture of the predicted RING and RING-like E3 ubiquitin ligases

Our datasets of *P. falciparum* and yeast RING and RING-like E3 ubiquitin ligases were searched for functional domains using SMART [100]. Our dataset from yeast was used as a reference.

## Supporting Information

Table S1 Exhaustive list of the proteins identified by the HMM search.
(0.37 MB XLS)

Table S2 Components of the Cdc48-Npl4-Ufd1 escort pathway in P. falciparum, predicted by BlastP, and their orthologs retrieved from http://orthomcl.cbil.upenn.edu
(0.04 MB XLS)

Table S3 Homologs of Skp1 in apicomplexan parasites.
(0.03 MB XLS)

Table S4 Protein dataset sources and release information.
(0.02 MB XLS)

Figure S1 By-domain dendrogram trees of the predicted apicomplexan proteins
(1.64 MB ZIP)
