# Supplementary figures and images for: Deciphering the Ubiquitin-Mediated Pathway in Apicomplexan Parasites: A Potential Strategy to Interfere with Parasite Virulence
Source: PLoS One. 2008 Jun 11;3(6):e2386. doi: 10.1371/journal.pone.0002386 (PMC2408969; doi:10.1371/journal.pone.0002386)

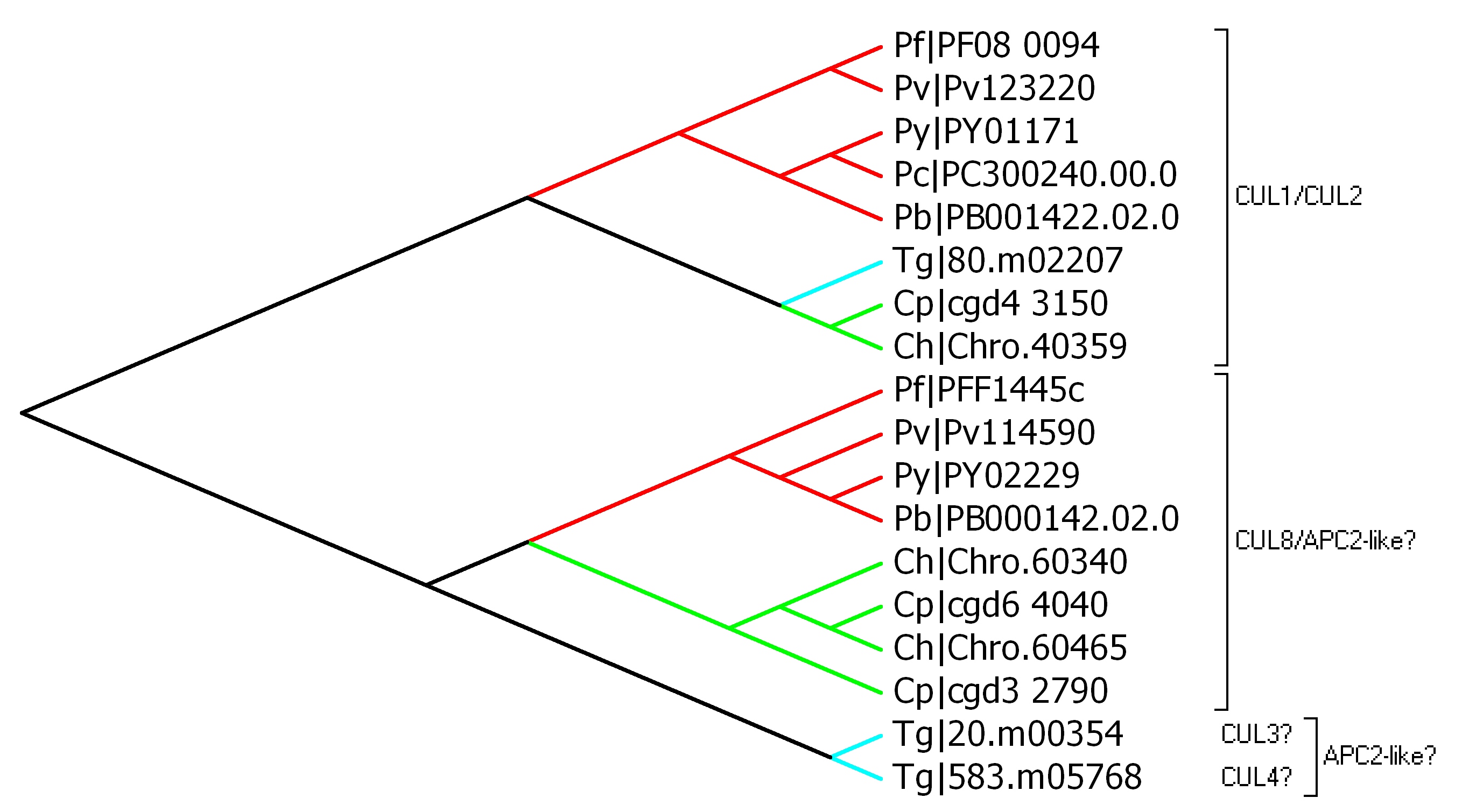

Supplement: Figure S1 — By-domain dendrogram trees of the predicted apicomplexan proteins (1.64 MB ZIP) [file pone.0002386.s005.zip › Supplemental_material_S4/Cullin-straight.TIF]

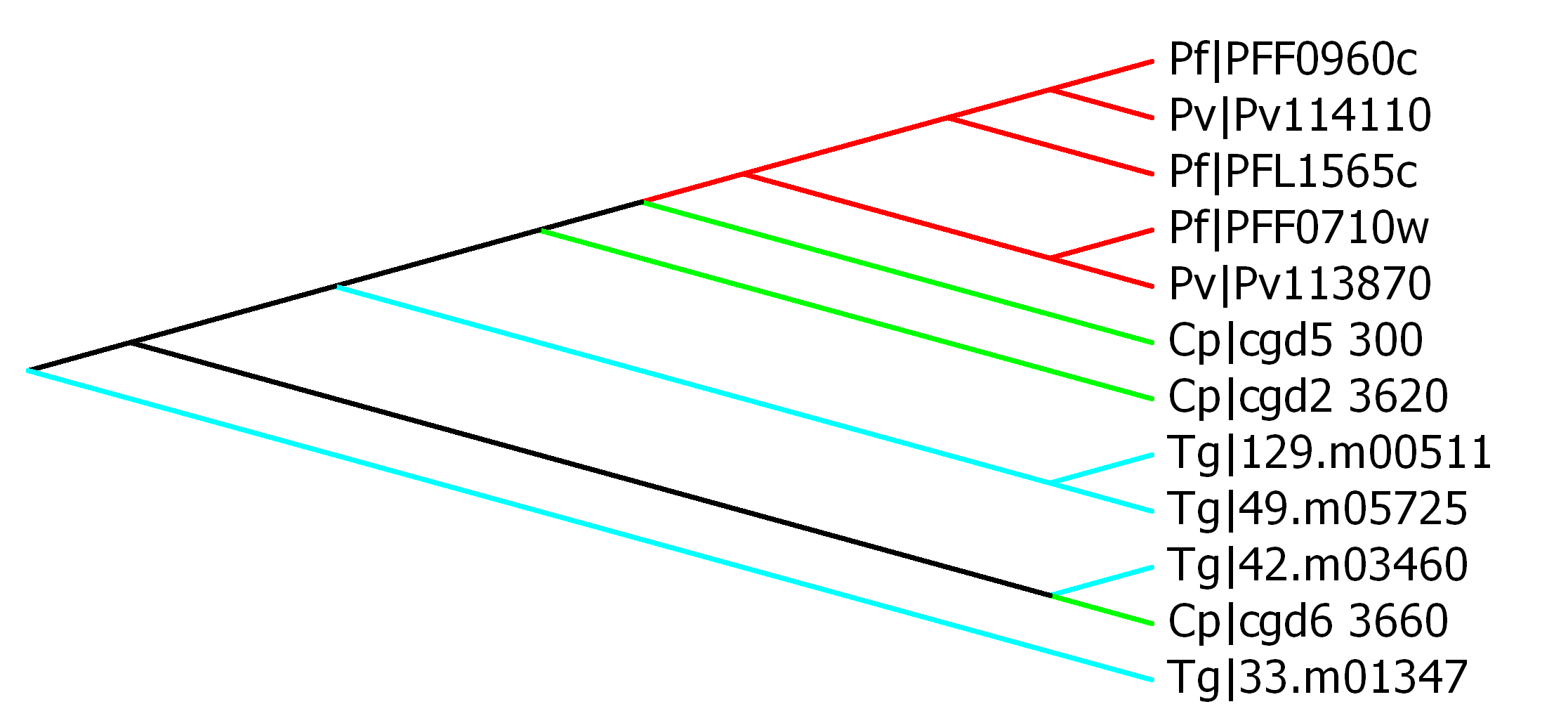

Supplement: Figure S1 — By-domain dendrogram trees of the predicted apicomplexan proteins (1.64 MB ZIP) [file pone.0002386.s005.zip › Supplemental_material_S4/F-box-straight.TIF]

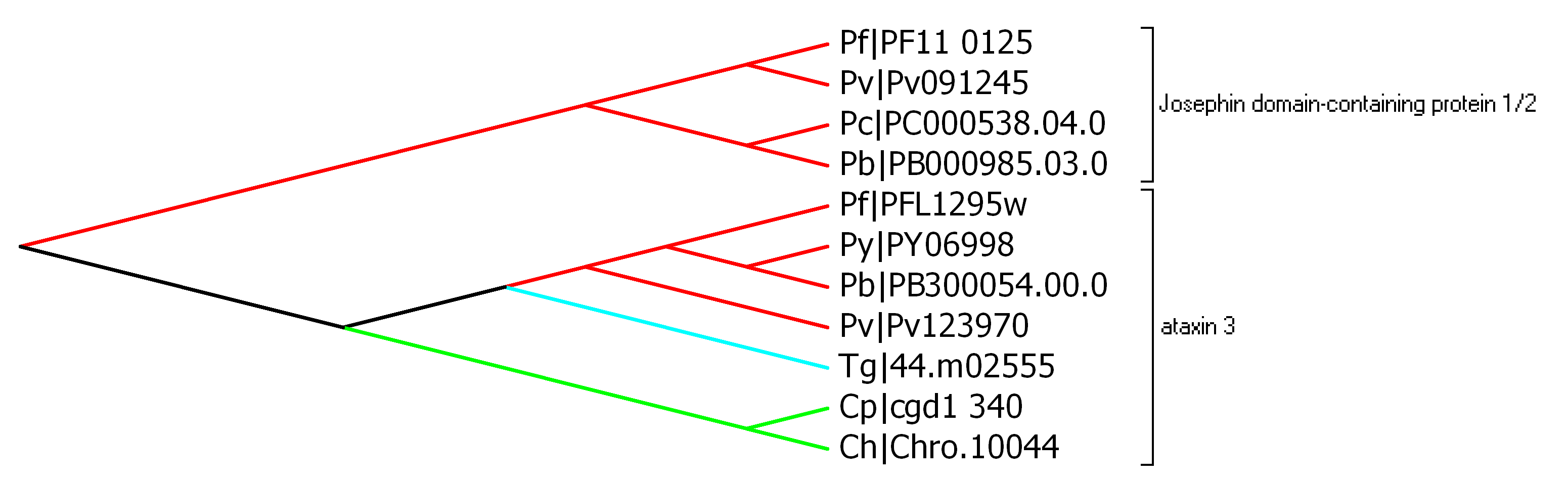

Supplement: Figure S1 — By-domain dendrogram trees of the predicted apicomplexan proteins (1.64 MB ZIP) [file pone.0002386.s005.zip › Supplemental_material_S4/Josephin-straight.TIF]

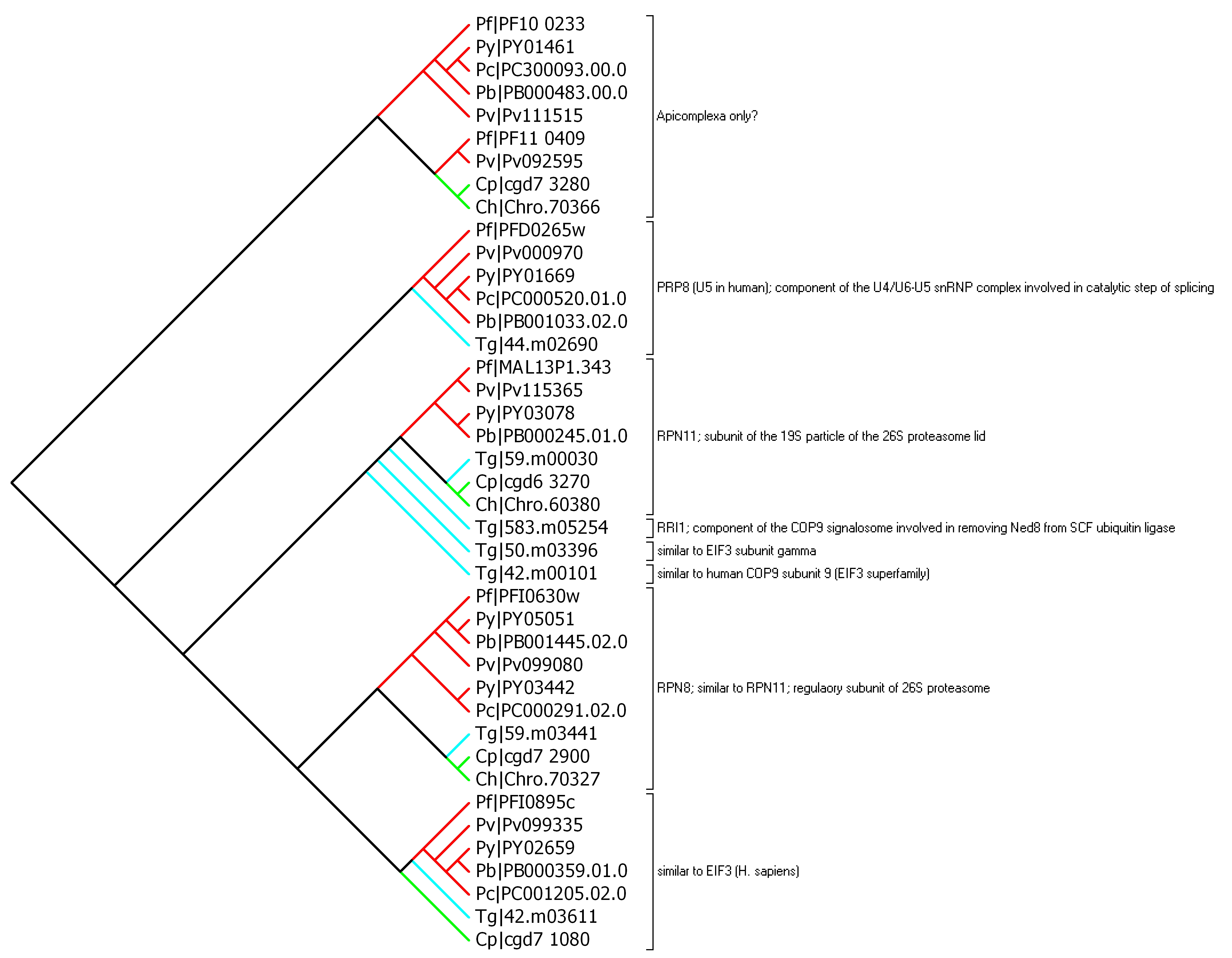

Supplement: Figure S1 — By-domain dendrogram trees of the predicted apicomplexan proteins (1.64 MB ZIP) [file pone.0002386.s005.zip › Supplemental_material_S4/Mov34-straight.TIF]

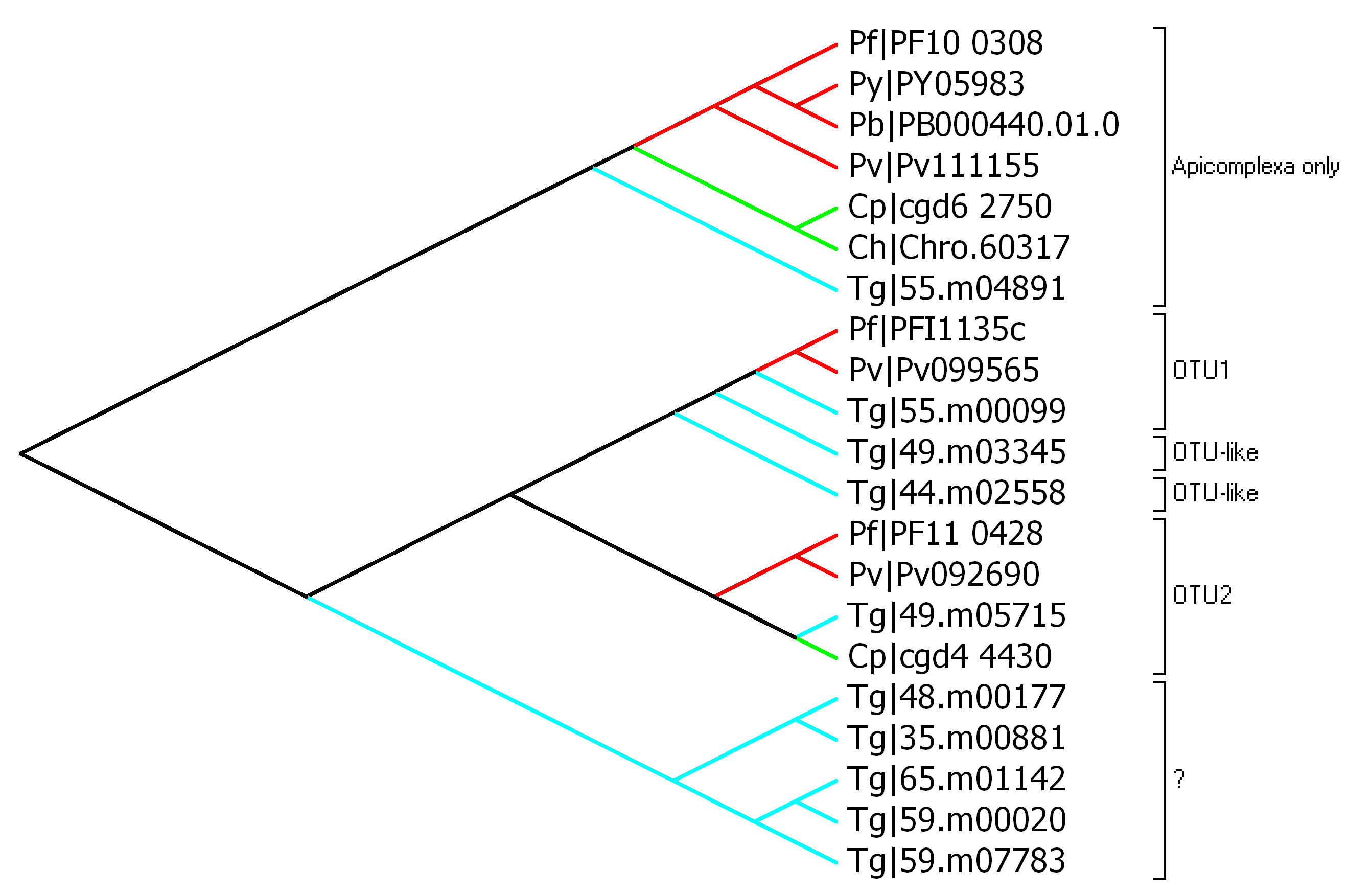

Supplement: Figure S1 — By-domain dendrogram trees of the predicted apicomplexan proteins (1.64 MB ZIP) [file pone.0002386.s005.zip › Supplemental_material_S4/OTU-straight.TIF]

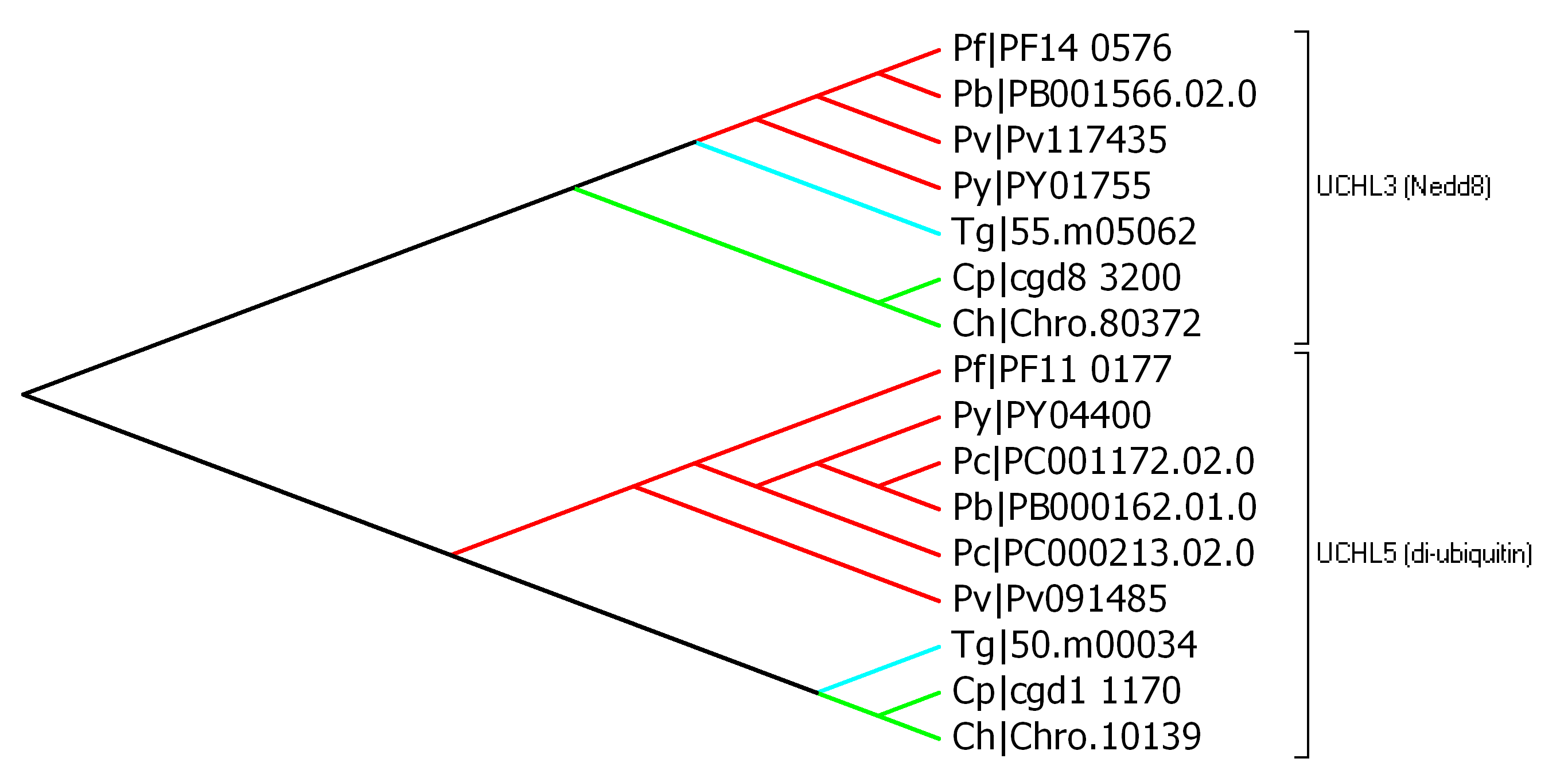

Supplement: Figure S1 — By-domain dendrogram trees of the predicted apicomplexan proteins (1.64 MB ZIP) [file pone.0002386.s005.zip › Supplemental_material_S4/Peptidase_C12-straight.TIF]

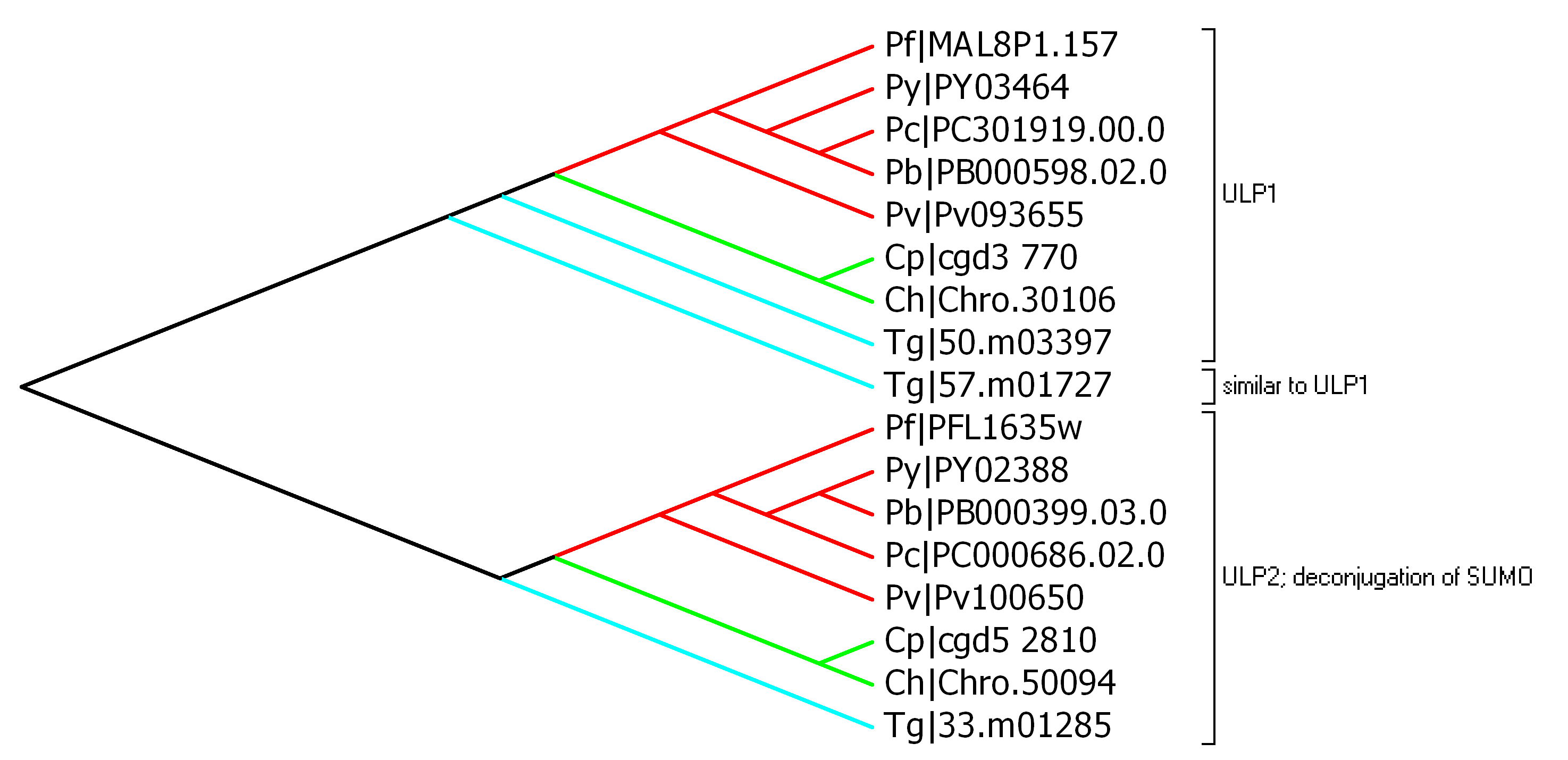

Supplement: Figure S1 — By-domain dendrogram trees of the predicted apicomplexan proteins (1.64 MB ZIP) [file pone.0002386.s005.zip › Supplemental_material_S4/Peptidase_C48-straight.TIF]

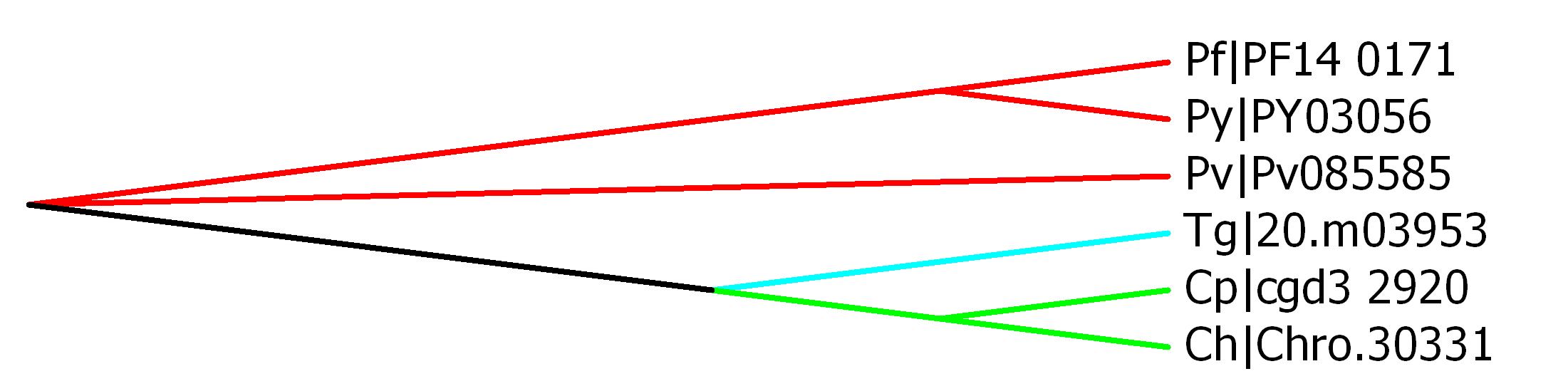

Supplement: Figure S1 — By-domain dendrogram trees of the predicted apicomplexan proteins (1.64 MB ZIP) [file pone.0002386.s005.zip › Supplemental_material_S4/Peptidase_C54-straight.TIF]

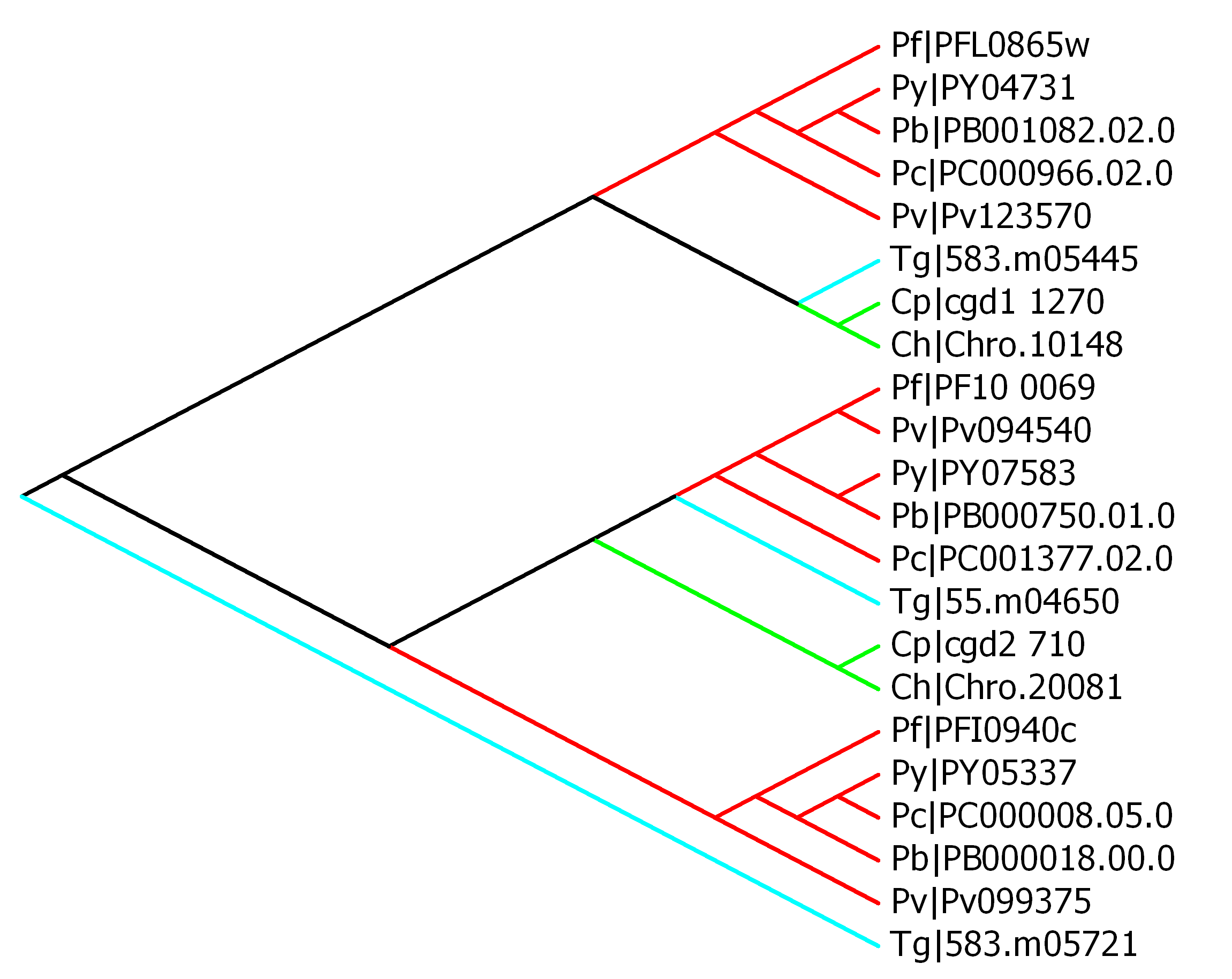

Supplement: Figure S1 — By-domain dendrogram trees of the predicted apicomplexan proteins (1.64 MB ZIP) [file pone.0002386.s005.zip › Supplemental_material_S4/PPPDE-straight.TIF]

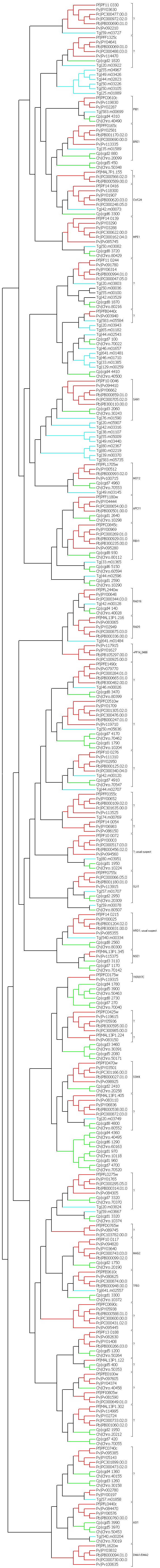

Supplement: Figure S1 — By-domain dendrogram trees of the predicted apicomplexan proteins (1.64 MB ZIP) [file pone.0002386.s005.zip › Supplemental_material_S4/RING-straight.TIF]

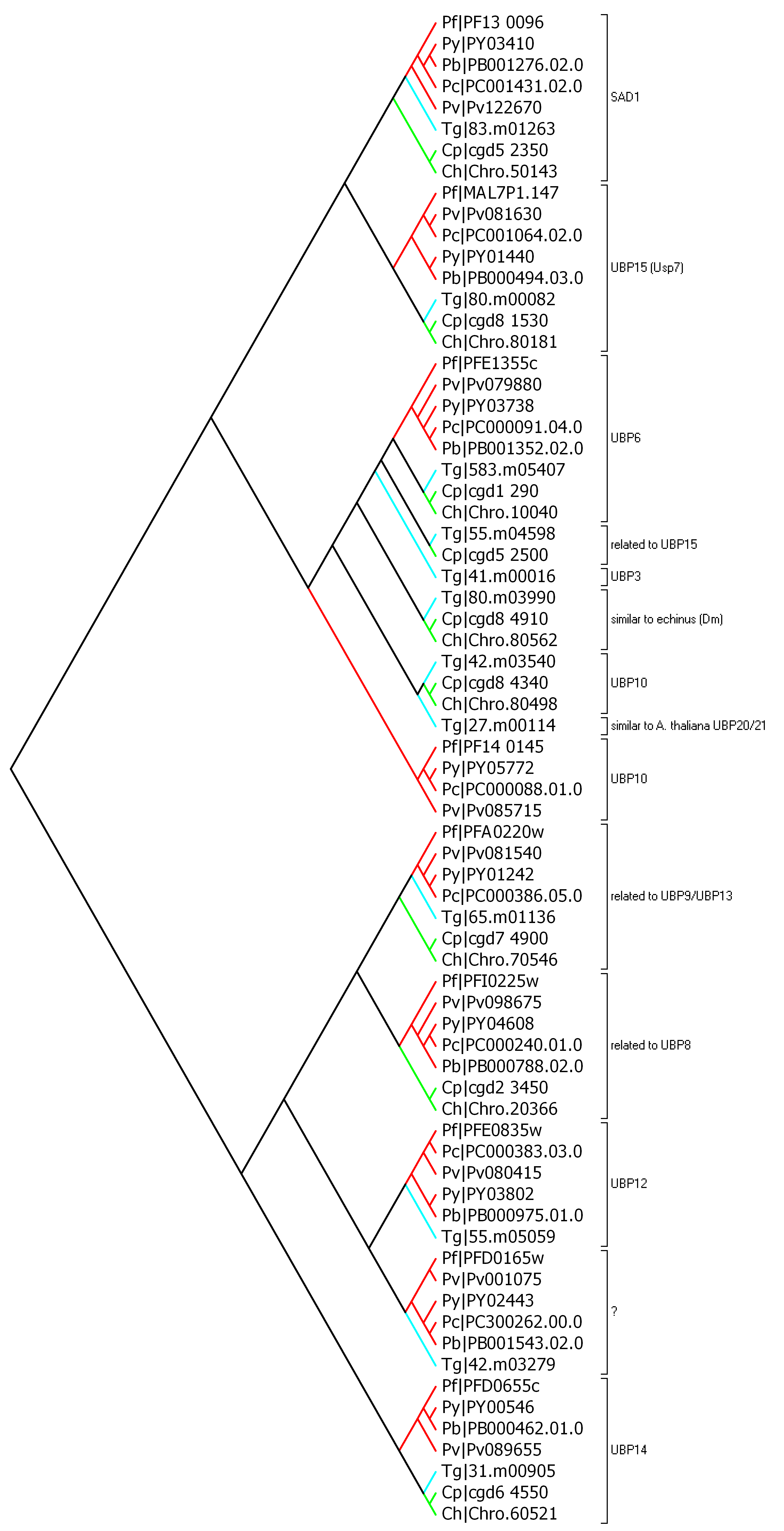

Supplement: Figure S1 — By-domain dendrogram trees of the predicted apicomplexan proteins (1.64 MB ZIP) [file pone.0002386.s005.zip › Supplemental_material_S4/UCH-straight.TIF]
